# Reconfiguration and loss of peritubular capillaries in chronic kidney disease

**DOI:** 10.1038/s41598-023-46146-4

**Published:** 2023-11-11

**Authors:** Charlotte Gaupp, Benjamin Schmid, Philipp Tripal, Aurélie Edwards, Christoph Daniel, Stefan Zimmermann, Margarete Goppelt-Struebe, Carsten Willam, Seymour Rosen, Gunnar Schley

**Affiliations:** 1 Department of Nephrology and Hypertension, Friedrich-Alexander University Erlangen-Nürnberg (FAU) and University Hospital Erlangen, Ulmenweg 18, 91054 Erlangen, Germany; 2 Optical Imaging Center Erlangen, Friedrich-Alexander University Erlangen-Nürnberg (FAU), Erlangen, Germany; 3 Department of Biomedical Engineering, Boston University, Boston, MA USA; 4 Department of Nephropathology, Friedrich-Alexander University Erlangen-Nürnberg (FAU) and University Hospital Erlangen, Erlangen, Germany; 5 Department of Computer Science, University of Applied Sciences Worms, Worms, Germany; 6 Department of Pathology, Beth Israel Deaconess Medical Center and Harvard Medical School, Boston, MA USA

**Keywords:** Chronic kidney disease, Chronic kidney disease, Confocal microscopy, Light-sheet microscopy

## Abstract

Functional and structural alterations of peritubular capillaries (PTCs) are a major determinant of chronic kidney disease (CKD). Using a software-based algorithm for semiautomatic segmentation and morphometric quantification, this study analyzes alterations of PTC shape associated with chronic tubulointerstitial injury in three mouse models and in human biopsies. In normal kidney tissue PTC shape was predominantly elongated, whereas the majority of PTCs associated with chronic tubulointerstitial injury had a rounder shape. This was reflected by significantly reduced PTC luminal area, perimeter and diameters as well as by significantly increased circularity and roundness. These morphological alterations were consistent in all mouse models and human kidney biopsies. The mean circularity of PTCs correlated significantly with categorized glomerular filtration rates and the degree of interstitial fibrosis and tubular atrophy (IFTA) and classified the presence of CKD or IFTA. 3D reconstruction of renal capillaries revealed not only a significant reduction, but more importantly a substantial simplification and reconfiguration of the renal microvasculature in mice with chronic tubulointerstitial injury. Computational modelling predicted that round PTCs can deliver oxygen more homogeneously to the surrounding tissue. Our findings indicate that alterations of PTC shape represent a common and uniform reaction to chronic tubulointerstitial injury independent of the underlying kidney disease.

## Introduction

Chronic kidney disease (CKD) is defined based on the presence of either kidney damage or decreased kidney function for three or more months, irrespective of its cause^1^. Typical histomorphological features of CKD are glomerulosclerosis and tubulointerstitial fibrosis. While the reduction in glomerular filtration rate (GFR) might direct attention to changes in the glomerular structure, it actually is the severity of tubulointerstitial damage in the kidney cortex rather than glomerular injury that correlates with kidney dysfunction in CKD^2,3^. Tubulointerstitial damage is widely regarded a final common pathway of CKD^4^. It comprises atrophy of renal tubules as well as an increase of the relative interstitial volume by accumulation of (myo-) fibroblasts, inflammatory cells and extracellular matrix^5^. Furthermore, functional and structural injury of the renal microvasculature is the consequence or even the cause of CKD development and progression^6,7^. The microvasculature of the kidney cortex consists of the glomerular capillaries and a second, postglomerular capillary network constituted by the peritubular capillaries which are in close alignment with cortical renal tubules^8^. Peritubular capillaries (PTCs) supply oxygen and nutrients to tubular and interstitial cells of the kidney cortex and are thus critical to their integrity. Capillary injury can ultimately lead to an irreversible loss of PTCs^9^. Accordingly, rarefaction of PTCs becomes a crucial limiting factor for the recovery of kidney tissue from injury^10,11^, and it correlates with the severity of kidney fibrosis in human patients and a variety of animal models^11–16^. Capillary rarefaction has been extensively studied by various, elaborate techniques^17,18^.

In addition to rarefaction, deformation of PTC shape was also reported in kidney fibrosis^19–22^. However, the alterations of PTC shape have not been thoroughly quantified. Therefore, we developed software-based algorithms for 2D and 3D semiautomatic segmentation and morphometric analysis of PTCs. PTC morphology was evaluated in three mouse models (adenine-induced tubulointerstitial nephritis, unilateral ureteral obstruction and long-term renal ischemia–reperfusion injury) and in human kidney biopsies with chronic tubulointerstitial injury. By comparison with healthy kidney tissue we aimed to define distinctive criteria of PTC shape for the classification of chronic tubulointerstitial injury. To address potential functional aspects of PTC shape, we established a computational model for the prediction of oxygen availability in the tissue surrounding PTCs as a function of their shape.

## Results

### Chronic tubulointerstitial injury leads to PTC loss and alterations of PTC shape

**Figure 1:**
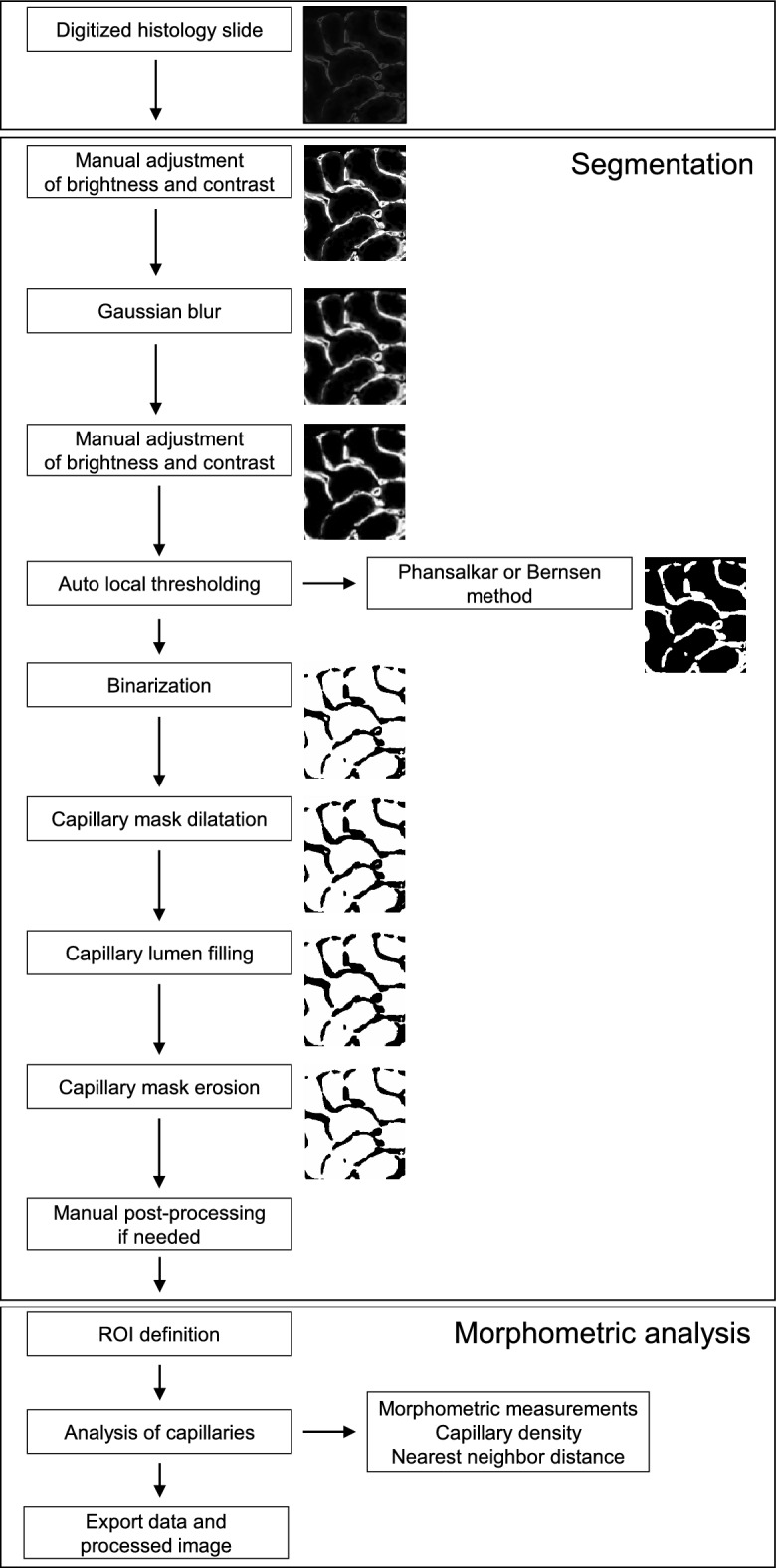
Procedure of 2D semiautomatic segmentation and morphometric analysis of peritubular capillaries performed in Fiji/ImageJ.

**Figure 2:**
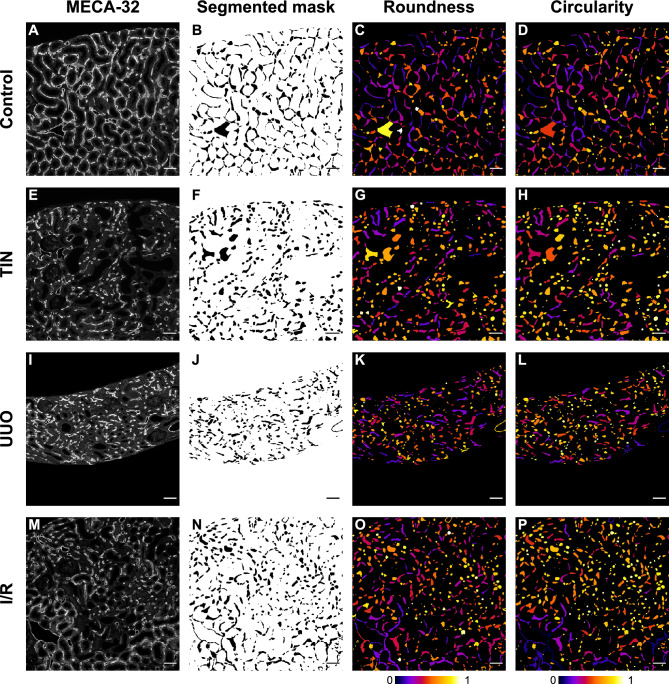
Peritubular capillaries in mouse models with chronic tubulointerstitial injury. Kidney sections from male C57BL/6NCrl control mice (n = 14) (**A**–**D**) and mice after 4 weeks fed an adenine-enriched diet (tubulointerstitial nephritis, TIN, n = 11) (**E**–**H**), 10 days after unilateral ureteral obstruction (UUO, n = 10) (**I**–**L**), and 14 days after bilateral renal ischemia–reperfusion (I/R) injury (n = 10) (**M**–**P**) were analyzed. (**A**, **E**, **I**, **M**) Representative MECA-32 immunostainings of peritubular capillaries. (**B, F, J, N**) Algorithm-based semiautomatic segmentation of MECA-32-stained peritubular capillaries (also see Fig. 1) combined with manual post-processing (also see Supplementary Fig. S2). Color-coded roundness (**C**, **G**, **K**, **O**) and circularity (**D**, **H**, **L**, **P**) values of peritubular capillaries. Color bars represent roundness and circularity values between 0 and 1. Scale bars represent 50 µm.

We analyzed PTC morphology in association with chronic tubulointerstitial injury in the kidney cortex of male C57BL/6NCrl mice subjected to adenine-induced tubulointerstitial nephritis (TIN), unilateral ureteral obstruction (UUO) and long-term renal ischemia–reperfusion (I/R) injury. Chronic tubulointerstitial injury, identified by interstitial fibrosis and tubular atrophy (IFTA) in H&E stainings, was widespread in the TIN and UUO and limited in the I/R model (Supplementary Fig. S1). Therefore, the whole kidney cortex was analyzed in TIN and UUO samples, while manually specified cortical regions were evaluated in I/R samples. PTCs were visualized by immunohistochemical staining for the endothelial cell marker MECA-32, segmented semiautomatically and evaluated morphometrically using a custom Fiji/ImageJ algorithm (Figs. 1, 2). As segmented PTCs were occasionally not correctly closed, connected or disconnected by the algorithm, single segmented PTCs were first manually post-processed when needed (Supplementary Fig. S2). Then, data obtained from post-processed and unprocessed segmented masks were compared, yielding essentially the same results, as explained below.

In comparison to healthy control kidneys, PTC area fraction declined significantly in kidneys with chronic tubulointerstitial injury, while the distance between adjacent PTCs increased significantly (Fig. 2, Supplementary Table S1). These data confirmed previous reports demonstrating loss of PTCs in response to chronic tubulointerstitial injury independent of the specific underlying disease.

**Figure 3:**
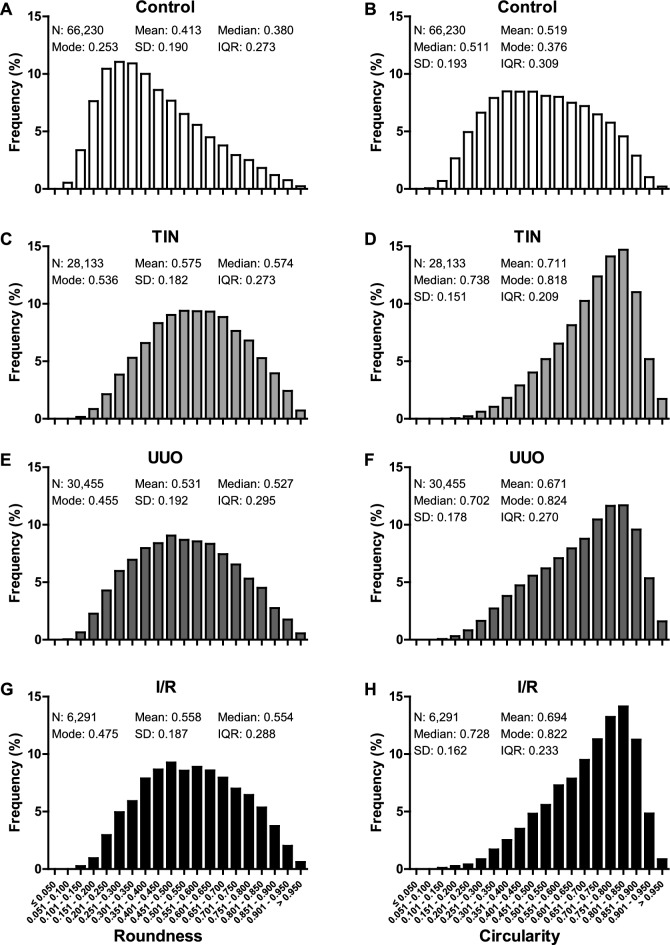
Roundness and circularity of post-processed peritubular capillaries in mouse models with chronic tubulointerstitial injury. Relative frequency distribution of all roundness (**A**, **C**, **E**, **G**) and circularity (**B**, **D**, **F**, **H**) values of peritubular capillaries in male C57BL/6NCrl control mice (n = 14) (**A**, **B**) and mice with adenine-induced tubulointerstitial nephritis (TIN, n = 11) (**C**, **D**), unilateral ureteral obstruction (UUO, n = 10) (**E**, **F**) and bilateral ischemia–reperfusion (I/R) injury (n = 10) (**G**, **H**). Semiautomatically segmented and manually post-processed capillary masks were analyzed. Indicated are the number (N) of capillaries, mean, median, mode, standard deviation (SD) and interquartile range (IQR) of distribution. Bar width represents ranges of 0.05.

In kidneys with chronic tubulointerstitial injury PTCs had a significantly reduced luminal area, perimeter, largest and shortest diameters in comparison to control kidneys (Fig. 2, Supplementary Table S1). PTC shape was variable ranging from elongated to round. In both healthy and diseased kidneys there were elongated as well as round PTCs, but more elongated capillary shapes clearly predominated in healthy kidneys, whereas more rounded capillary shapes prevailed in kidneys with chronic tubulointerstitial injury (Fig. 2). This was reflected in higher mean, median, and modal values of the shape parameters roundness and circularity in kidneys with chronic tubulointerstitial damage in comparison to healthy kidneys (Fig. 3, for the definition of parameters see Methods section).

In kidneys with chronic tubulointerstitial injury, the absolute as well as the relative number of round PTCs (defined by roundness ≥ 0.47 and circularity ≥ 0.60 respectively, as determined by the Youden index in ROC analysis) increased significantly by a factor of about 2 (Supplementary Table S2). Hence, chronic tubulointerstitial injury was not only associated with loss of PTCs, but also with distinct changes of PTC shape which were uniform and consistent in all mouse models.

Analysis of unprocessed segmented PTCs showed essentially the same results as obtained for manually post-processed segmented PTCs (Supplementary Fig. S3, Supplementary Table S3), suggesting that manually post-processing of PTC masks is dispensable in future morphometric analyses.

PTCs with altered shape were scattered in the kidney cortex and were not clearly located near a specific tubule segment, as shown by co-immunofluorescence staining for MECA-32 and markers for proximal tubules, distal tubules or collecting ducts in control kidneys and exemplary in TIN (Supplementary Fig. S4).

### Tissue oxygenation is more homogenous adjacent to round PTCs

**Figure 4:**
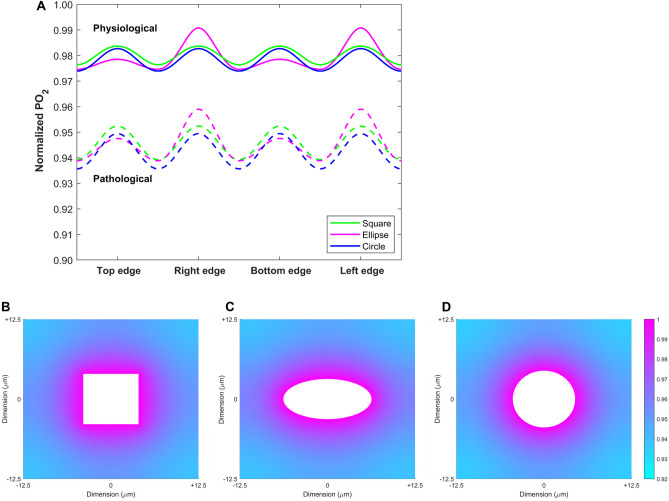
Oxygen delivery from a peritubular capillary into the surrounding tissue depending on its shape. (**A**) Predicted values of the partial pressure of oxygen (PO_2_) along the edges of one periodic unit cell, relative to PO_2_ at the capillary surface. Results are shown for three different, idealized capillary cross-sections: square, ellipse, and circle. The cross-sectional area of each capillary is fixed at 80 µm^2^ in the physiological case and at 62.5 µm^2^ in the pathological case. The size of the unit cell (i.e., the distance between the centers of 2 adjacent capillaries) is 20 µm in the physiological case and 25 µm in the pathological case. (**B**–**D**) Predicted PO_2_ profile in the tissue surrounding a peritubular capillary in one unit cell under pathological conditions. The capillary cross-section is represented as either a square (**B**), an ellipse (**C**), or a circle (**D**). The PO_2_ values are normalized by the PO_2_ at the capillary surface. The length of the unit cell is 25 µm and capillary density is 0.10 for all shapes.

To analyze a possible functional role of PTC shape, we established a computational model to assess tissue oxygenation depending on PTC shape. We calculated the partial pressure of oxygen (PO_2_) in the tissue surrounding a capillary with a circular, elliptical or square cross-section for a physiological state (characterized by lower nearest neighbor distance and higher capillary density) and a pathological state (with higher nearest neighbor distance and lower capillary density). Geometrical parameters used in the computational model are summarized in Supplementary Table S4. According to the model, tissue PO_2_ levels in the kidney cortex were lower in the pathological than in the physiological condition, and the average PO_2_ value was rather independent of PTC shape (Fig. 4A). However, the rounder the capillary was, the more homogenous was the oxygen tension in the surrounding tissue (Fig. 4B-D). Thus, our computational model suggested that alterations in PTC shape can improve the homogeneity of kidney tissue oxygenation in chronic tubulointerstitial injury.

### 3D light sheet fluorescence microscopy demonstrates microvascular simplification and reconfiguration in chronic tubulointerstitial injury

**Figure 5:**
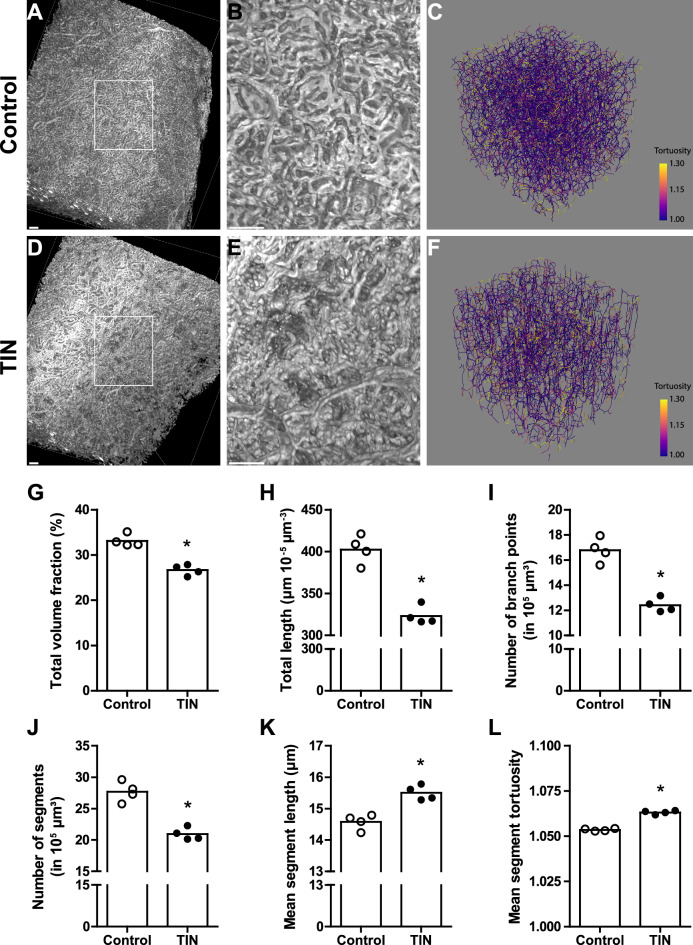
3D analysis of the kidney cortex microvasculature in murine chronic tubulointerstitial nephritis. Male C57BL/6NCrl control mice (n = 4) (**A**–**C**) and mice with adenine-induced tubulointerstitial nephritis (TIN, n = 4) (**D**–**F**) were intravenously injected with an Alexa Fluor® 647-conjugated anti-CD31 antibody to visualize the vascular endothelium. After optical tissue clearing whole mouse kidneys were imaged by light sheet fluorescence microscopy. 3D reconstructions (**A**, **B**, **D**, **E**) and 3D skeletons (**C**, **F**) of capillaries in the kidney cortex were generated. The square areas in **A**, **D** are magnified in **B**, **E** respectively. Scale bars represent 100 µm (**A**, **D**), 400 µm (**B**, **E**). Skeletonized capillaries were color-coded according to their tortuosity. Sample volume is 391 × 419 × 400 µm^3^ (**C**, **F**). In skeletonized 3D image reconstructions the total volume fraction (**G**) and total length of the capillary network (**H**), the number of capillary branch points (**I**) and segments (**J**) as well as the mean segment length (**K**) and mean segment tortuosity (**L**) were quantified. *p < 0.05 exact two-sided Mann–Whitney U test.

To complement the 2D histology findings, the microvasculature from control mice and mice with TIN was labelled using an intravitally injected anti-CD31 antibody, and whole kidneys were imaged by light sheet fluorescence microscopy. After 3D reconstruction, skeletonized capillaries in the kidney cortex were analyzed (Fig. 5A–F, Supplementary Video S1-S4). Whereas control mice had a dense and complex cortical capillary network, the microvascular bed was simplified in mice with TIN. This was reflected in a substantially lower total volume fraction and shorter total length of the capillary network. Furthermore, numbers of capillary branch points and segments were reduced, while the mean capillary segment length and tortuosity increased (Fig. 5G–L, Supplementary Fig. S5). Thus, in vivo fluorescence labeling of the renal microvasculature did not only reveal its significant loss but also its simplification and reconfiguration in response to chronic tubulointerstitial injury.

### Circularity of PTCs correlates with kidney dysfunction and tubulointerstitial injury in human patients

Next, we examined PTCs in human kidney biopsies from renal allografts and from patients with IgA nephropathy with different severity of chronic tubulointerstitial injury and kidney dysfunction (Supplementary Table S5, Supplementary Fig. S6). Within each biopsy, regions with chronic tubulointerstitial injury and/or preserved (“control”) kidney tissue were manually specified in H&E stainings. PTCs were visualized by CD31 immunofluorescence and confocal laser microscopy.

**Figure 6:**
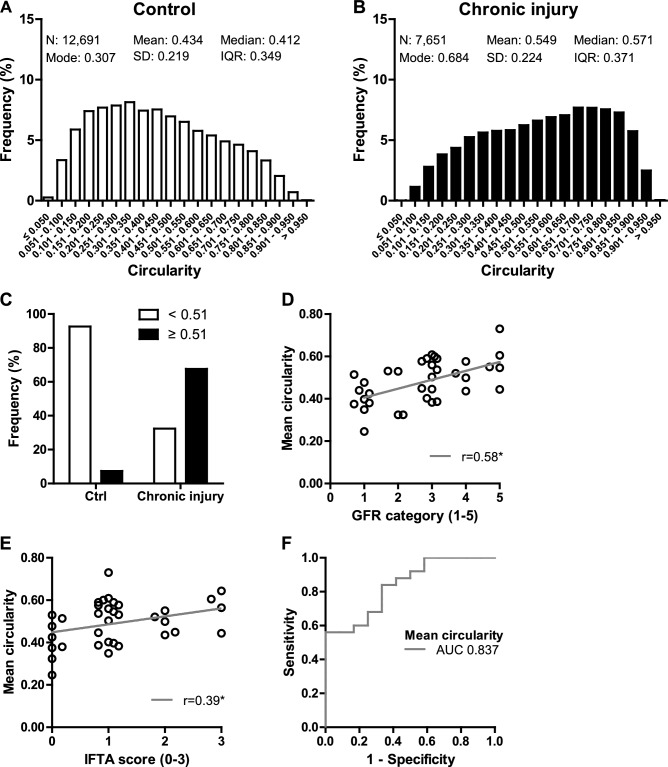
Circularity of peritubular capillaries in human kidney biopsies with chronic tubulointerstitial injury. Human kidney biopsies from renal allografts (n = 10) and patients with IgA nephropathy (n = 30) were stained for CD31. In each kidney biopsy, regions with normal parenchyma (control) and/or regions with chronic tubulointerstitial injury were manually selected. Semiautomatically segmented, unprocessed peritubular capillaries (PTCs) were evaluated morphometrically in control regions (n = 27) and regions with chronic tubulointerstitial injury (n = 37). For analysis all values per region (**A**, **B**), mean values per biopsy and region (**C**) or mean values per biopsy (**D**–**F**) were used. (**A**, **B**) Relative frequency distribution of all PTC circularity values in control regions (**A**) and regions with chronic tubulointerstitial injury (**B**). Indicated are the number (N) of capillaries, mean, median, mode, standard deviation (SD) and interquartile range (IQR) of distribution. Bar width represents ranges of 0.05. **(C)** The optimal cut-off value of PTC mean circularity (0.51) to distinguish preserved kidney tissue from chronic tubulointerstitial injury was determined by the Youden index in receiver operating characteristic (ROC) analysis. Relative frequency of PTC mean circularity values less (white bars) and equal or greater (black bars) than the cut-off value in control regions and regions with chronic tubulointerstitial injury. (**D**, **E**) Linear regression between PTC mean circularity and glomerular filtration rate (GFR) categories (G1-G5 according to the KDIGO classification) (**D**) and between PTC mean circularity and interstitial fibrosis and tubular atrophy (IFTA) grades (0–3 according to the Banff classification) (**E**). The linear regression coefficient r is indicated. *p < 0.05 two-tailed t-test. (**F**) The ability of PTC mean circularity to classify the presence of chronic kidney disease (eGFR < 60 ml/min/1.73 m^2^) or interstitial fibrosis and tubulointerstitial injury (IFTA score > 1) was assessed by ROC analysis. The area under the ROC curve (AUC) is indicated.

Chronic tubulointerstitial injury in human kidney biopsies was associated not only with a significant loss of PTCs but also with a significant reduction in luminal area, perimeter and diameters in comparison to control kidney tissue (Supplementary Table S6). In regions with chronic tubulointerstitial injury the distribution of PTC circularity (Fig. 6A,B) shifted to higher mean, median and modal values, reflecting the predominance of rounder PTC shapes in kidney regions with chronic tubulointerstitial injury compared with control regions. Thus, the morphometric analyses in human kidney biopsies were consistent with the results in the mouse models.

Round PTCs (defined by mean circularity ≥ 0.51, as determined by the Youden index in ROC analysis) were more frequent by a factor of 9 in regions with chronic tubulointerstitial injury than in control regions (Fig. 6C). PTC mean circularity correlated significantly with GFR categories and IFTA scores (Fig. 6D,E, Supplementary Table S7). Both, low GFR and IFTA, are important risk factors for CKD progression. Furthermore, PTC mean circularity predicted the presence of CKD or IFTA (AUC 0.837) (Fig. 6F, Supplementary Table S8).

Our findings demonstrate that chronic tubulointerstitial injury, not only in mouse models but also in human patients, is associated with significant changes in the shape of PTCs. In particular, round PTC shapes predominate in kidneys with chronic tubulointerstitial injury. PTC mean circularity correlated with established parameters of kidney function and kidney damage and classified the occurrence of CKD or IFTA, suggesting that the alteration of PTC shape may be of diagnostic significance in kidney disease.

## Discussion

Functional and structural damage to the renal microcirculation, specifically to the network of PTCs, has emerged as a key factor in the development as well as the progression of CKD. In this study, we quantified the shape of PTCs in mouse models and human kidney biopsies with chronic tubulointerstitial injury by using a custom Fiji/ImageJ algorithm for the semiautomatic segmentation and morphometric analysis of PTCs.

In association with chronic tubulointerstitial injury, the shape of PTCs remarkably changed. Whereas in normal kidney tissue PTCs were predominantly elongated, most PTCs had a rounder shape in regions with chronic tubulointerstitial injury. In line with our findings, Johnson et al. described distorted capillary morphology, with shrinking and rounding of capillaries in rats with microvascular injury and tubulointerstitial fibrosis induced by catecholamine infusion^19^. Furthermore, deformed PTCs in fibrotic lesions were observed in rat models of glomerulonephritis, obstructive nephropathy, 5/6 nephrectomy as well as human chronic allograft nephropathy^20–23^. However, PTC morphology was not quantified in these studies. Morphometric analysis demonstrated significantly reduced luminal area, perimeter, diameters and significantly increased circularity and roundness of PTCs in association with chronic tubulointerstitial injury. These alterations of PTC morphology were consistent in three mouse models as well as in human kidney biopsies.

Our data, obtained in mouse and human kidney tissue with different disease pathologies, suggest that changes of PTC shape occur frequently and uniformly in association with chronic tubulointerstitial injury. Therefore, they are probably an integral component of the pathomechanism of chronic tubulointerstitial injury. To date, the cellular and molecular mechanisms of these changes remain unknown.

The (mean) circularity represented a robust parameter to describe the shape of PTCs. It correlated significantly with eGFR categories and IFTA grades and classified the presence of CKD or IFTA in human patients. To the best of our knowledge, this is the first study which used PTC shape to characterize the degree of kidney injury. As lower eGFR and IFTA are common important risk factors for CKD progression to end-stage kidney disease/kidney replacement therapy^2,24–26^, PTC shape may be predictive of kidney outcomes. However, this needs to be confirmed in prospective cohort studies.

Quantitative morphometry of microvessels has been used to study tumor (lymph-) angiogenesis and has been correlated with tumor invasiveness, (lymph node) metastasis, prognosis or response to systemic therapy in solid and hematologic malignancies^27–34^. These associations were particularly found for more irregularly shaped microvessels. Thus, morphometric parameters related to the size and particularly to the shape of microvessels may add important diagnostic and prognostic information in patients not only with malignancies but also with CKD.

The software-based algorithm for the semiautomatic segmentation and morphometric analysis of renal capillaries used in this study is relatively simple and practical, can be easily modified and applied to the analysis of microvascular morphology in other tissues and organs, and can probably be established in most laboratories. The present algorithm still requires the users’ manual intervention, but can presumably be modified to work fully automatically which could accelerate workflow and reduce user error as well as intra- and inter-observer variability. In recent years, deep learning-based algorithms have improved biomedical image segmentation^35,36^. In kidney research, deep learning has been used to identify renal histological structures^37–41^ and to quantify interstitial fibrosis, tubular atrophy and glomerulosclerosis^42–44^ and inflammatory infiltration^45^. Therefore, implementing deep learning-based segmentation of PTCs in future analyses could enhance speed and accuracy in comparison to classical segmentation methods.

PTC morphology was examined not only with 2D but also with 3D histology. Classically, serial tissue sections were aligned and reconstructed into 3D images^46^. Recent improvements in tissue clearing techniques in combination with optical sectioning by confocal, multiphoton or light sheet microscopy, allow faster 3D visualization of intact tissues, organs or even whole organisms^47^. Here, we performed immunostaining of whole-mount kidney microvessels in vivo, using an intravenously injected fluorescent-labelled anti-CD31 antibody, followed by a tissue clearing procedure and light sheet fluorescence microscopy. This technique revealed not only a significant rarefaction (as also demonstrated by 2D histology) but, more importantly, a substantial structural simplification and reconfiguration of the renal capillary network in association with chronic tubulointerstitial injury. As part of structural vascular reconfiguration, tortuosity of PTCs was increased significantly in chronic tubulointerstitial injury. This finding is in line with previous studies reporting increased tortuosity of preglomerular arterioles in mice after I/R injury and UUO as well as in poststenotic human kidneys using micro-computed tomography or ultrasound super-resolution imaging^48–50^.

To assess the impact of PTC shape on tissue oxygenation, we implemented a two-dimensional model of oxygen diffusion and consumption. In the classical Krogh cylinder model, a single cylindrical capillary is surrounded by a coaxial cylindrical region of tissue. While different contours of tissue regions have been examined, to the best of our knowledge the role of capillary shape has not been studied. The computational model predicted that under physiological and pathological conditions the shape of capillary cross-section (circular, elliptical or square) has a minimal effect on average tissue PO_2_ levels in the kidney cortex. This is probably due to the fact that the kidney cortex is still relatively well-oxygenated even in pathological situations^51^, and other parameters (e.g. capillary density and surface area) have a greater influence on tissue PO_2_. However, the oxygenation of the kidney tissue surrounding a capillary with a round cross-section was predicted to be more homogeneous. Thus, round PTC shape per se can improve the homogeneity of kidney tissue oxygenation. The significance of this observation requires further experimental investigation.

In summary, our study demonstrates alterations of capillary shape associated with chronic tubulointerstitial injury which also affect tissue oxygenation. We provide methods and first evidence that PTC shape could be an additional parameter for assessing the severity of CKD which should be investigated in further studies using fully automated segmentation and morphometric analysis. Elucidating the dynamics and mechanisms responsible for the alterations of PTC shape will improve our understanding of CKD pathogenesis.

## Methods

### Animal experiments

All animal experiments were approved by the animal care and use committee of local government authorities (Regierung von Mittelfranken, Ansbach, Germany; Az 54-2532.1-6/11, 54-2532.1-8/11, 54-2532.1-11/13) and were conducted in strict accordance with the Guide for the Care and Use of Laboratory Animals of the National Institutes of Health and the ARRIVE guidelines^52,53^.

Male C57BL/6NCrl mice, weighing 20 to 25 g, were obtained from Charles River (Sulzfeld, Germany). Animals were housed under a 12:12 h light–dark cycle at constant temperature (22 ± 1 °C) and humidity (55 ± 5%) with free access to tap water and standard rodent chow (V1534-000, ssniff Spezialdiäten GmbH, Soest, Germany), unless otherwise stated.

Tubulointerstitial nephritis (TIN) was induced by feeding mice (n = 16) an adenine-enriched diet (0.2% w/w; ssniff Spezialdiäten GmbH) for 4 weeks^54,55^. To induce bilateral renal ischemia–reperfusion (I/R) injury (n = 10), both renal pedicles were occluded for 25 min with atraumatic microaneurysm clamps (FE 723 K, Aesculap, Tuttlingen, Germany)^56,57^. For unilateral ureteral obstruction (UUO) (n = 10), the left mid-ureter was double-ligated with two 4–0 silk sutures and cut between the ligations^58^. Surgery was performed under isoflurane anesthesia and pre-emptive analgesia with buprenorphine (0.05 to 0.1 mg/kg s.c.). 14 days after I/R injury and 10 days after UUO, respectively, mice were sacrificed by exsanguination under deep isoflurane anesthesia. Healthy male C57BL/6NCrl mice served as controls (n = 19).

Subgroups of control mice (n = 4) and mice with TIN (n = 4) were injected intravenously with 10 µl Alexa Fluor® 647-conjugated rat anti-mouse CD31 antibody (CD31-AF647, 102516; BioLegend, San Diego, CA; diluted 1:15 in phosphate-buffered saline) or phosphate-buffered saline in the tail vein 45 min before sacrifice.

After euthanasia, mice were successively perfused transcardially with 6% hydroxyethyl starch (Fresenius Kabi, Bad Homburg, Germany) containing 0.5 g/l procaine (Steigerwald, Darmstadt, Germany), Jonosteril (Fresenius Kabi) and 4% paraformaldehyde at 150 cm H_2_O of perfusion pressure. Kidneys were removed and post-fixed by immersion with 4% paraformaldehyde, before being embedded in paraffin.

### Human kidney samples

The studies involving human participants were approved by the local ethics committee (Ethik-Kommission der Friedrich-Alexander-Universität Erlangen-Nürnberg, Ref.-No. 4415) and carried out according to the Declaration of Helsinki^59^. Informed consent was obtained from all participants.

To investigate morphological alterations of the microvasculature in human kidneys, formalin-fixed paraffin-embedded biopsy samples from patients with IgA nephropathy (n = 30) and renal allograft biopsies (n = 10) were selected. GFR was calculated using the 4-variable modified Modification of Diet in Renal Disease (MDRD) equation^60^, and GFR values were categorized according to the 2012 Kidney Disease: Improving Global Outcomes (KDIGO) CKD guideline^1^. Histology of IgA nephropathy was evaluated according to the updated Oxford Classification^61^. Interstitial fibrosis and tubular atrophy were scored semiquantitatively according to the Banff classification^62^.

### Immunofluorescence staining of renal microvessels

Mouse and human kidney tissues were sectioned using a rotation microtome (Microm, HM340E, Thermo Fisher Scientific, Waltham, MA). Paraffin sections (2 µm) were dewaxed in xylol and rehydrated in graded alcohol series.

Hematoxylin and eosin (H&E) staining (1.05174 and HT110132, Sigma-Aldrich, Taufkirchen, Germany) was performed using standard protocols. Slides were mounted with Entellan (1.07960, Sigma-Aldrich). Consecutive slices were processed for immunohistochemistry.

Antigen retrieval was performed in target retrieval solution (pH 6, S1699, Dako, Glostrup, Denmark) for 5 min using a pressure cooker.

Mouse kidney sections were incubated with a rat monoclonal anti-MECA-32 antibody (Developmental Studies Hybridoma Bank, Iowa City, IA; diluted 1:2 in antibody diluent, S0809, Dako) overnight at 4 °C, followed by a Cy3-conjugated donkey polyclonal anti-rat IgG antibody (712-165-153, Jackson ImmunoResearch, Ely, United Kingdom; diluted 1:500 in Tris-buffered saline with 1% bovine serum albumin) for 2 h at room temperature.

For co-immunofluorescence staining of MECA-32 and renal tubule segment-specific markers in mouse kidneys the following antibodies were used: mouse monoclonal anti-megalin (sc-515772, Santa Cruz Biotechnology, Santa Cruz, CA, 1:50), rabbit polyclonal anti-rat sodium chloride cotransporter (NCC, SLC12A3) (SPC-402, StressMarq Biosciences, Victoria, BC, Canada, 1:500), sheep polyclonal anti-rat 11β-hydroxysteroid dehydrogenase type 2 (11βHSD2) (AB1296, Chemicon International, Temecula, CA, 1:250), Alexa Fluor™ 488 goat polyclonal anti-mouse IgG (A-11001, Thermo Fisher Scientific, 1:250), Alexa Fluor™ 488 goat polyclonal anti-rabbit IgG (A-11034, Thermo Fisher Scientific, 1:250), Cy3 donkey polyclonal anti-sheep IgG antibody (713-165-147, Jackson ImmunoResearch, 1:250), Alexa Fluor™ 488 goat polyclonal anti-rat IgG (A-11006, Thermo Fisher Scientific, 1:250). All antibodies were diluted in DAKO antibody diluent.

MECA-32 (PLVAP, PV-1) is specifically expressed in endothelial cells not only of blood but also of lymphatic vessels. Recently, Prox-1 (Prospero homeobox protein 1)-positive lymphatic endothelial cells were determined to account for 0.05% of all cells in the adult mouse kidney^63^. Therefore, we assumed that MECA-32 + endothelial cells represent blood vessels without a significant contribution of lymphatic vessels.

Results were confirmed with an anti-CD31 antibody in human kidney biopsies. Human kidney sections were subsequently blocked with avidin/biotin block (SP-2001, Vector Laboratories, Burlingame, CA), peroxidase block (S2023, Dako) and serum-free protein block (X0909, Dako). Then, slides were incubated with a mouse monoclonal anti-human CD31 antibody (M0823, Dako; diluted 1:200 in antibody diluent, Dako) overnight at 4 °C, followed by a biotinylated goat polyclonal anti-mouse IgG antibody (BP-9200, Vector Laboratories), avidin solution (Vectastain Elite ABC Kit, PK-6100, Vector Laboratories) and tyramide signal amplification solution (TSA Plus Biotin Kit, NEL749A, Akoya Biosciences, Hopkinton, MA) for 30 min each at room temperature. Signals from CD31-positive endothelial cells were visualized with Streptavidin-Cy3 conjugate (SA-1300, Vector Laboratories; diluted 1:200 in antibody diluent, Dako). All slides were mounted with Fluoromount (F4680, Sigma-Aldrich).

Negative controls for immunostaining were performed by substituting the primary antibodies with an equivalent concentration of normal rat or mouse serum.

### Mouse 2D immunofluorescence imaging

One kidney section from each mouse was investigated. In each immunostained kidney section, 10–23 consecutive non-overlapping fields covering the complete kidney cortex were captured at 200× magnification. Photographs were taken using a DS-Qi2 digital camera (Nikon Instruments, Tokyo, Japan) attached to an Eclipse 80i microscope (Nikon Instruments) and an Intensilight C-HGFI illuminator (Nikon Instruments).

### Human 2D immunofluorescence imaging

Confocal imaging of CD31-stained total human kidney biopsies was performed on a Zeiss Spinning Disc Axio Z1 live cell observer (Zeiss, Jena, Germany). Samples were imaged with a 25× magnification lens (LCI Plan Apochromat 25x/0.8, Zeiss) and oil as immersion medium. The scan area was adapted according to the size of the biopsy section, and tiles were stitched with ZEN blue 2.3 software (Zeiss). The fluorophore Cy3 was excited at 561 nm wave length with a laser intensity of 50%, and emission was recorded between 598 and 660 nm at a dynamic range of 14 bit.

Corresponding H&E-stained human tissue samples were digitized by using a VENTANA DP 200 Slide Scanner (Roche Diagnostics, Rotkreuz, Switzerland).

### Semiautomatic segmentation of capillaries in 2D images

A semiautomatic image segmentation method was developed in Fiji/ImageJ (version 1.52; National Institutes of Health, Bethesda, MD) to quantify capillary size and shape^64^. The workflow is based on published segmentation approaches and is summarized in Fig. 1^65^. A grayscale image was manually adjusted for brightness and contrast to enhance the visibility of the capillary outline. Next, a Gaussian filter (σ = 2.5) was applied to smooth the capillary outline. As the Gaussian filter might not preserve image brightness, the image was manually adjusted for brightness and contrast again. Then, the capillaries were thresholded by using the “Auto Local Threshold” function with the Bernsen (r = 35 pixels, for mouse TIN, UUO, I/R samples and all human biopsies) or Phansalkar method (r = 35 pixels, for mouse control samples) resulting in a binary mask of capillaries. The segmented capillaries were successively dilated, filled and eroded to obtain capillaries with a continuous, uninterrupted outline and filled lumen without distorting their size and shapes.

Occasionally, the algorithm resulted either in discontinuity and fragmentation of the segmented capillaries, due to variations in staining intensity of the thin vascular endothelium, or in overlap of adjacent capillaries. Thus, if needed, segmented capillaries were manually closed, connected or disconnected as appropriate, using a Wacom Intuos Pro tablet (Wacom Technology Corporation, Portland, OR) (Supplementary Fig. S2). Capillary masks from mouse tissue were analyzed with and without manual post-processing. Capillary masks from human kidney samples were not manually processed.

To determine the adequacy of the segmentation algorithm, outlines of segmented capillaries were overlaid on the original immunofluorescence image, as exemplarily shown in Supplementary Fig. S7.

### Morphometric analysis of segmented capillary masks

Regions of interest (ROI) with chronic tubulointerstitial injury were manually specified in H&E stainings and transferred onto the segmented capillary masks. Within these ROIs, capillaries (size 10–8000 µm^2^) were analyzed using the “Analyze Particles” command in Fiji/ImageJ. The following parameters of size were measured: area, perimeter, width and height of bounding box, major and minor diameter of ellipse, maximum and minimum Feret diameter (Supplementary Fig. S8). These parameters of shape were calculated: bounding box ratio = width/height of bounding box, aspect ratio = major/minor diameter of ellipse, Feret ratio = maximum/minimum Feret diameter, roundness = 4*area/(π*major diameter of ellipse^2^), circularity (also known as shape factor) = 4π*area/perimeter^2^^66^. Roundness indicates the elongation of a particle and varies from approaching 0 for an elongated particle to 1 for an equiaxed particle. A circularity value of 1 indicates a perfect circle, and the more an object lengthens, the more its circularity tends toward 0.

Capillaries were color-coded based on their roundness and circularity values by using the BioVoxxel Toolbox (BioVoxxel, Ludwigshafen, Germany).

Capillary density was determined as total area fraction or total volume fraction with respect to the total area or total volume of interest^48^.

To calculate the mean distance between capillaries in binary masks, a custom Fiji/ImageJ plugin was used. Briefly, capillaries were separated using connected components labeling. Labels were then used as seeds for an Euclidean distance-based watershed algorithm to assign each background pixel to the closest vessel. In the resulting compartments, vessel neighborhoods were identified as two neighbor pixels being assigned to different vessels, and shortest distances were calculated for all neighbor pairs.

To determine how many PTCs need to be analyzed to detect significant changes in roundness or circularity of PTCs between control and injured kidney tissue, the required sample size was estimated at the end of the study using G*Power (version 3.1.9.6)^67^ and the following assumptions: α error 0.05, β error 0.10, means and standard deviations from Figs. 3, 6 and Supplementary Fig. S3. Analysis of less than 90 PTCs was required to detect significant differences in capillary shape between healthy and injured kidney tissue.

### Light sheet fluorescence microscopy and 3D image reconstruction

Light sheet fluorescence microscopy (LSFM) was performed on kidneys from healthy mice (n = 4) and mice with TIN (n = 4), as described previously by Klingberg et al.^68^ To differentiate valid endothelial fluorescence from potential autofluorescence and fluorescence signal of non-endothelial cells, two additional animals (one from each subgroup) did not receive the Alexa Fluor® 647-conjugated anti-CD31 antibody and served as antibody-negative controls. Kidneys were fixed by transcardial perfusion and post-fixed by immersion with 4% paraformaldehyde, dehydrated in increasing concentrations of ethanol (pH 9) and cleared with ethyl cinnamate (112372; Sigma-Aldrich) for 14 hours^68^. The cleared kidneys were imaged using a LaVision BioTec UltraMicroscope II Light Sheet Microscope (LaVision BioTec, Bielefeld, Germany), including an Olympus MVX10 Zoom Microscope Body (Olympus, Tokyo, Japan), LaVision BioTec Laser Module and an Andor Neo sCMOS Camera (Oxford Instruments, Belfast, United Kingdom). The fluorophore Alexa Fluor® 647 was excited at 640 nm, and emissions were detected using a 680/30 nm filter. Kidney autofluorescence was determined at an excitation wavelength of 488 nm. Light sheet thickness (z-plane) was set to 5 µm for diseased and 4 µm for control samples. Z-stacks consisting of serial 2D images were 3D rendered using the Fiji/ImageJ plugin “3Dscript” (Supplementary Videos S1, S2)^69^.

Renal microvessels were segmented using a custom-written Fiji/ImageJ plugin for interactive thresholding based on the eigenvalues of the pixel-wise calculated hessian matrix (https://github.com/bene51/Interactive_Hessian_Segmentation, date of last access: 2023 Feb 1)^70^. Then, image stacks were resized in z-direction using linear interpolation to obtain isotropic volumetric data. Elongated structures were subsequently emphasized using the Fiji/ImageJ “Tubeness” filter plane-by-plane (σ = 4 µm), followed by global thresholding (t = 100). The resulting binary images were skeletonized using the Fiji/ImageJ plugin “Skeletonize (2D/3D)”. For each voxel on the skeleton, the vessel thickness was estimated by investigating its local neighborhood in the tubeness-filtered image. These vessel skeletons were analyzed with the Fiji/ImageJ plugin “Analyze Skeleton (2D/3D)” and VesselVio^71,72^. Vessel tortuosity was calculated as the ratio between the total length of a vessel segment and the Euclidean distance (shortest linear distance) between its end points. This ratio equals 1 for a straight line and is infinite for a circle. The vessel skeletons were visualized using VesselVio (Supplementary Videos S3, S4).

### Computational model of oxygen delivery from peritubular capillaries

To examine the impact of capillary shape on tissue oxygenation, we developed a simplified computational model of two-dimensional oxygen (O_2_) diffusion and consumption in the tissue surrounding peritubular capillaries. We assumed evenly spaced capillaries and defined an idealized, periodic unit centered on one capillary, termed unit cell. In cross-section, the capillary was represented as either a circle, an ellipse (with an aspect ratio of 2), or a square, all with the same cross-sectional area. The surrounding tissue was taken to be homogenous. Partial oxygen pressure (PO_2_) in the tissue was computed by solving the following O_2_ diffusion–reaction equation using MATLAB® (version R2022a, MathWorks, Natick, MA):

$${\alpha }_{O2}{D}_{O2}{\nabla }^{2}{PO}_{2}={R}_{O2}$$

$${PO}_{2}={P}_{0}\, \mathrm{at\, the\, surface\, of\, the\, capillary}$$

$${\nabla PO}_{2}=0 \,\mathrm{at\, the \,edge\, of \,the \,unit \,cell\, }(\mathrm{by \,symmetry})$$

where $\nabla$ is the gradient operator, α_O2_ and D_O2_ are the solubility and diffusivity of O_2_ in the surrounding tissue, respectively, and R_O2_ is the volumetric rate of O_2_ consumption, assumed to be zero order. We used the following parameter values: α_O2_ = 1.53 × 10^–6^ mol O_2_ L^-1^ mmHg^-1^, D_O2_ = 2.4 × 10^–5^ cm^2^ s^-1^ and R_O2_ = 13.2 × 10^–5^ mol O_2_ L^-1^ s^-1^^73–75^. The partial pressure of O_2_ in the capillary, P_0_, was set to 45 mmHg^75^.

In the physiological case, the nearest neighbor distance (i.e., the length L of the unit cell) was set to 20 µm and the capillary cross-section to 80 µm^2^, yielding a density of 80 µm^2^/(20 µm × 20 µm) = 0.20. In the pathological case, L was set to 25 µm and the capillary cross-section to 62.5 µm^2^, yielding a density of 62.5 µm^2^/(25 µm × 25 µm) = 0.10. Values for the average nearest neighbor distance and capillary density are based on our measurements in Supplementary Table S1.

### Statistical analyses

Data analysis was performed using IBM SPSS Statistics for Windows version 21.0 (IBM, Armonk, NY) and GraphPad Prism version 5.04 for Windows (GraphPad Software, La Jolla, CA). All data are presented as mean ± SD.

Kruskal–Wallis H test with Dunn-Bonferroni post hoc test and two-sided Mann–Whitney U test were used to determine statistical significance, as appropriate.* P* < 0.05 was considered statistically significant.

Diagnostic performance of morphological parameters was assessed using Receiver Operation Characteristic (ROC) curves and corresponding area under the curve (AUC).

Correlations were assessed using Spearman's correlation coefficient. Statistical significance was tested by two-tailed t-test.

### Supplementary Information

Supplementary Information 1.

Supplementary Video 1.

Supplementary Video 2.

Supplementary Video 3.

Supplementary Video 4.

## Data Availability

All data generated or analyzed during this study are included in this published article and its Supplementary Information files.
