# Supplementary material for: Reconfiguration and loss of peritubular capillaries in chronic kidney disease
Source: Sci Rep. 2023 Nov 11;13:19660. doi: 10.1038/s41598-023-46146-4 (PMC10640592; doi:10.1038/s41598-023-46146-4)
Supplement: Supplementary file 1 — Supplementary Information 1. [file 41598_2023_46146_MOESM1_ESM.pdf]

Supplementary Information

Reconfiguration and loss of peritubular capillaries in chronic kidney disease

Charlotte Gaupp [1], Benjamin Schmid [2], Philipp Tripal [2], Aurélie Edwards [3], Christoph Daniel [4], Stefan Zimmermann [5], Margarete Goppelt-Struebe [1], Carsten Willam [1], Seymour Rosen [6], Gunnar Schley [1]

[1] Department of Nephrology and Hypertension, Friedrich-Alexander University Erlangen-Nürnberg (FAU) and University Hospital Erlangen, Erlangen, Germany

[2] Optical Imaging Center Erlangen, Friedrich-Alexander University Erlangen-Nürnberg (FAU), Erlangen, Germany

[3] Department of Biomedical Engineering, Boston University, Boston, MA

[4] Department of Nephropathology, Friedrich-Alexander University Erlangen-Nürnberg (FAU) and University Hospital Erlangen, Erlangen, Germany

[5] Department of Computer Science, University of Applied Sciences Worms, Worms, Germany

[6] Department of Pathology, Beth Israel Deaconess Medical Center and Harvard Medical School, Boston, MA

Supplementary Figure S1: Chronic tubulointerstitial injury in mouse models.

Representative hematoxylin and eosin (H&E) staining of kidney sections from male C57BL/6NCrl control mice (n=14) (**A**) and mice after 4 weeks fed an adenine-enriched diet (tubulointerstitial nephritis, TIN, n=11) (**B**), 10 days after unilateral ureteral obstruction (UUO, n=10) (**C**), and 14 days after bilateral renal ischemia-reperfusion (I/R) injury (n=10) **(D)**. Scale bars represent 100 µm.

Supplementary Figure S2: Semiautomatic segmentation of peritubular capillaries in combination with manual post-processing.

(**A, D**) Original immunofluorescence images of MECA-32-stained peritubular capillaries in mouse kidney cortex.

(**B, E**) Capillary masks after semiautomatic segmentation in Fiji/ImageJ using the algorithm shown in **Figure 1**. Irregular and insufficient staining intensity of the capillary endothelium can lead to incomplete segmentation.

(**C, F**) Capillary masks were manually closed and/or connected as needed before morphometric analysis.

Scale bars represent 15 µm.

Supplementary Figure 3: Roundness and circularity of unprocessed peritubular capillaries in mouse models with chronic tubulointerstitial injury.

Relative frequency distribution of all roundness (**A, C, E, G**) and circularity (**B, D, F, H**) values of peritubular capillaries in male C57BL/6NCrl control mice (n=14) (**A, B**) and mice with adenine-induced tubulointerstitial nephritis (TIN, n=11) (**C, D**), unilateral ureteral obstruction (UUO, n=10) (**E, F**) and bilateral ischemia-reperfusion (I/R) injury (n=10) (**G, H**). Semiautomatically segmented, unprocessed masks were analyzed. Indicated are the number (N) of capillaries, mean, median, mode, standard deviation (SD) and interquartile range (IQR) of distribution. Bar width represents ranges of 0.05.

Supplementary Figure S4: Co-localization of peritubular capillaries with distinct renal tubule segments in mouse kidneys.

Kidney sections from male C57BL/6NCrl control mice (**A, C, E**) and mice with adenine-induced tubulointerstitial nephritis (TIN) (**B, D, F**) were co-stained with antibodies against MECA-32 and megalin (marker for proximal tubule) (**A, B**), sodium chloride cotransporter (NCC, marker for distal convoluted tubule) (**C, D**) or 11β-hydroxysteroid dehydrogenase type 2 (11βHSD2, marker for distal convoluted tubule and collecting duct) (**E, F**).

Scale bars represent 50 µm.

Supplementary Figure S5: Capillary tortuosity in control mouse kidneys and chronic tubulointerstitial nephritis.

Three-dimensional (3D) skeletons of kidney cortex microvasculature were generated using light sheet fluorescence microscopy in male C57BL/6NCrl control mice (n=4) (**A**) and mice with adenine-induced tubulointerstitial nephritis (TIN, n=4) (**B**). Per animal 12-31 3D vascular skeletons were analyzed.

Relative frequency distribution of mean capillary segment tortuosity per 3D vascular skeleton. Indicated are the number (N) of 3D skeletons, mean, median, mode, standard deviation (SD), and interquartile range (IQR) of capillary segment tortuosity. Bar width represents ranges of 0.00125.

Supplementary Figure S6: Chronic tubulointerstitial injury and microvascular alterations in human kidney biopsies.

Human kidney biopsies from patients with IgA nephropathy (**A-D**) and from time-zero renal allograft (**E, F**) were stained with hematoxylin and eosin (H&E) (**A, C, E**) and immunohistochemically for CD31 (**B, D, F**). Images show mild (**A, B)**, moderate (**C, D**) and severe (**E, F)** chronic tubulointerstitial injury and alterations of the renal microvasculature. Scale bars represent 200 µm.

Supplementary Figure S7: Adequacy of the segmentation algorithm.

Peritubular capillaries were stained for MECA-32 (**A, D**), segmented semiautomatically and post-processed manually (**B, E**), as shown in **Figure 1** and **Supplementary Figure S2**. To assess the adequacy of the segmentation algorithm, outlines (yellow) of the segmented masks were overlaid on the original immunofluorescence images (**C, F**).

Scale bars represent 50 µm.

Supplementary Figure S8: Parameters of morphometric analysis.

Peritubular capillaries were segmented semiautomatically using the algorithm in **Figure 1** and evaluated morphometrically using the "Analyze Particles" command in Fiji/ImageJ. The depicted parameters were measured.

Modified from: https://imagej.net/plugins/biovoxxel-toolbox, retrieved August 7, 2021.

Scale bar represents 15 µm.

Supplementary Video 1: Three-dimensional (3D) reconstruction of the kidney cortex microvasculature of a healthy mouse.

Reconstructed 3D microvasculature of the kidney cortex of a healthy control mouse using light sheet fluorescence microscopy (also see **Figure 5A, B**). Scale bar represents 100 µm.

Supplementary Video 2: Three-dimensional (3D) reconstruction of the kidney cortex microvasculature in murine chronic tubulointerstitial nephritis.

Reconstructed 3D microvasculature of the kidney cortex of a mouse with adenine-induced chronic tubulointerstitial nephritis using light sheet fluorescence microscopy (also see **Figure 5D, E**). Scale bar represents 100 µm.

Supplementary Video 3: Three-dimensional (3D) vascular skeleton of a healthy mouse.

Skeletonized 3D reconstruction of the kidney cortex microvasculature of a healthy control mouse (also see **Figure 5C**). Skeletonized capillaries were color-coded according to their

tortuosity. Color bar indicates tortuosity values between 1.0 and 1.3. Sample volume is 391 x 419 x 400 μm^3.

Supplementary Video 4: Three-dimensional (3D) vascular skeleton in murine chronic tubulointerstitial nephritis.

Skeletonized 3D reconstruction of the kidney cortex microvasculature of a mouse with adenine-induced chronic tubulointerstitial nephritis (also see **Figure 5F**). Skeletonized capillaries were color-coded according to their tortuosity. Color bar indicates tortuosity values between 1.0 and 1.3. Sample volume is 391 x 419 x 400 μm^3.

Supplementary Figure S1

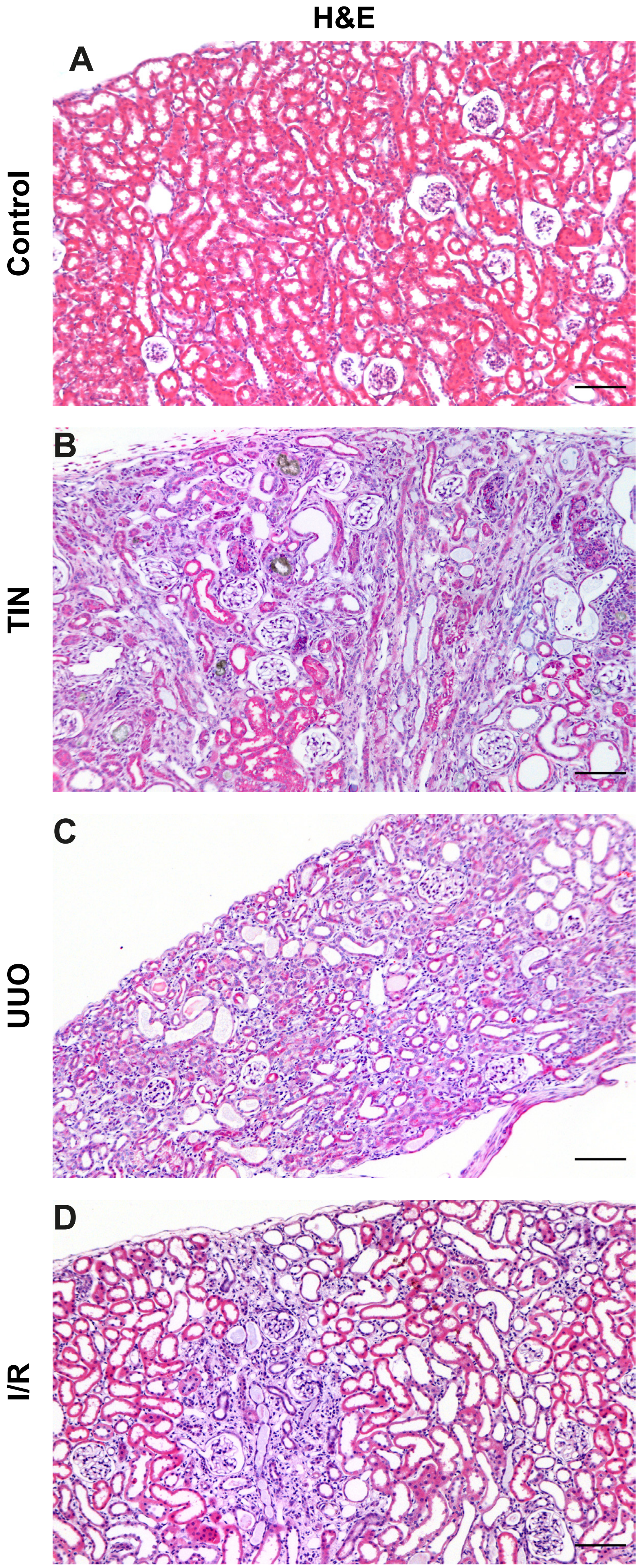

Supplementary Figure S2

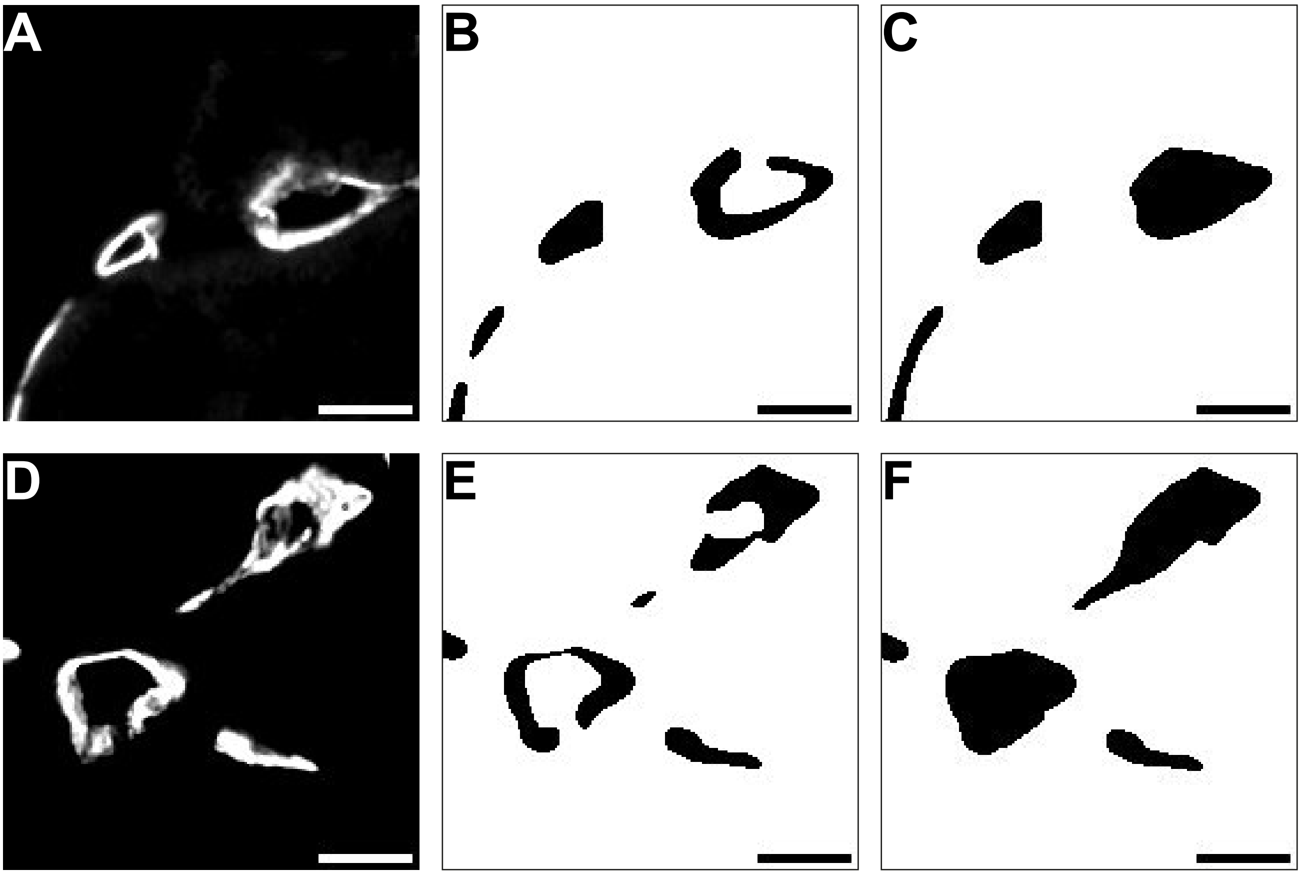

Supplementary Figure S3

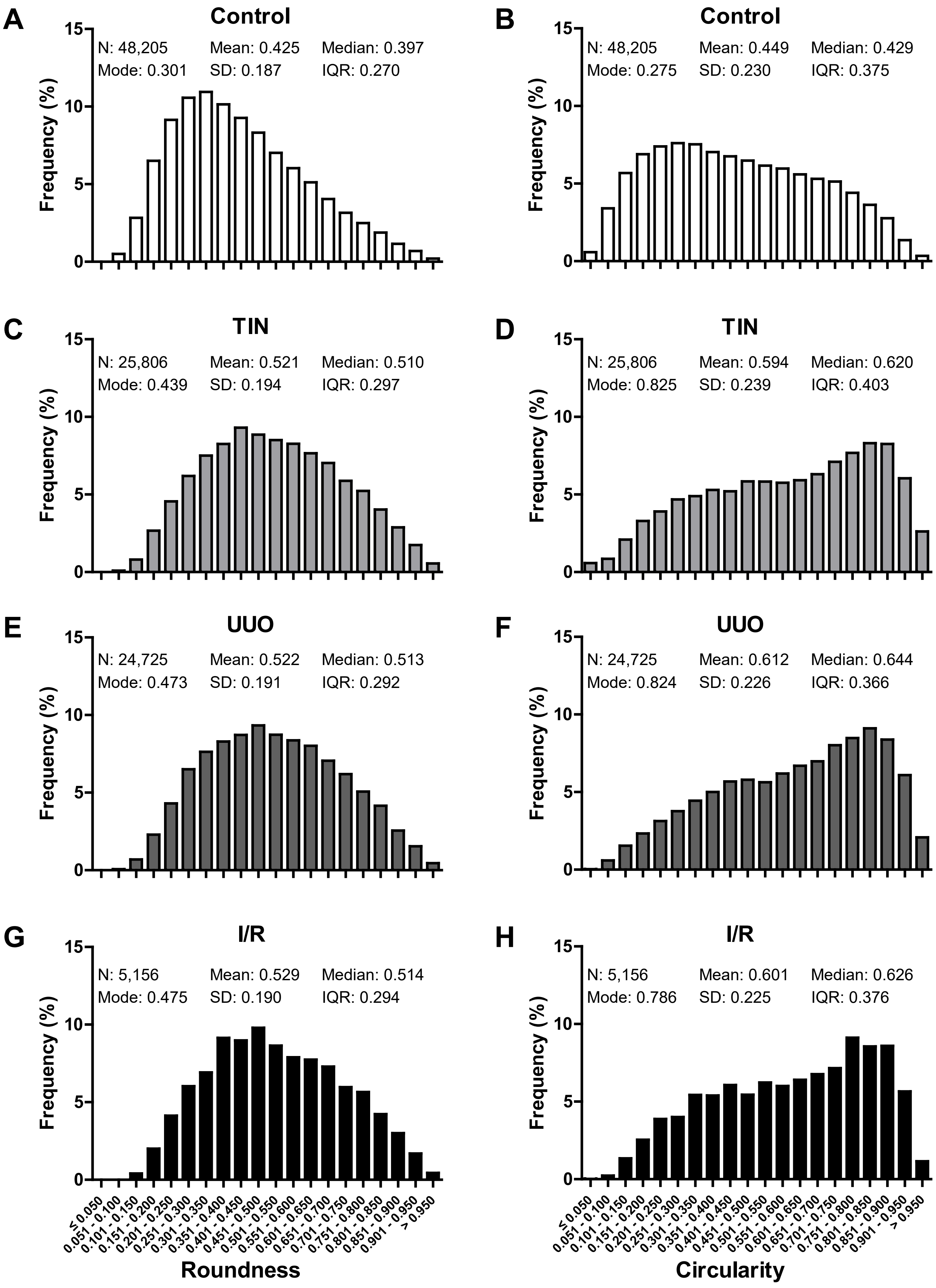

Supplementary Figure S4

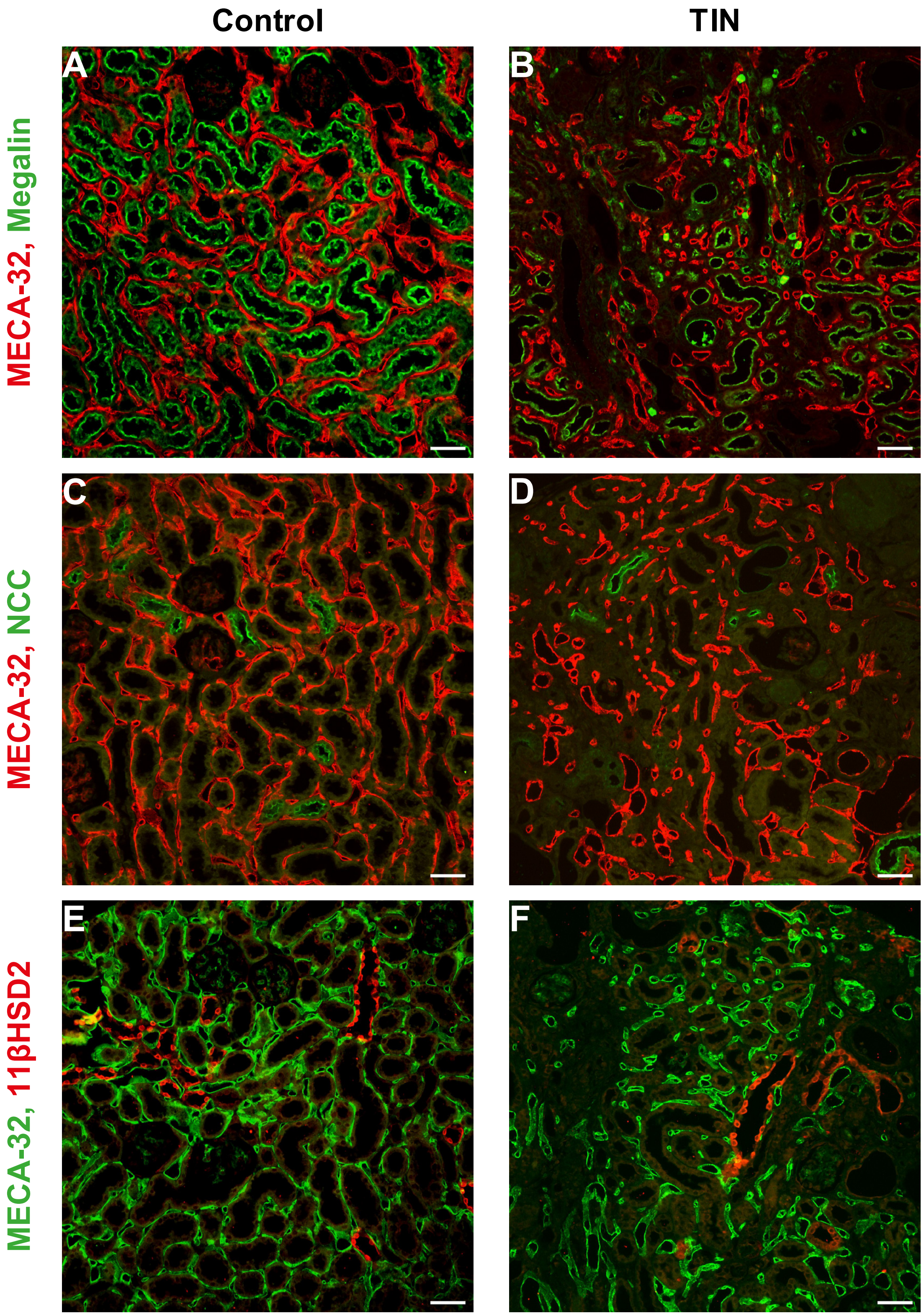

Supplementary Figure S5

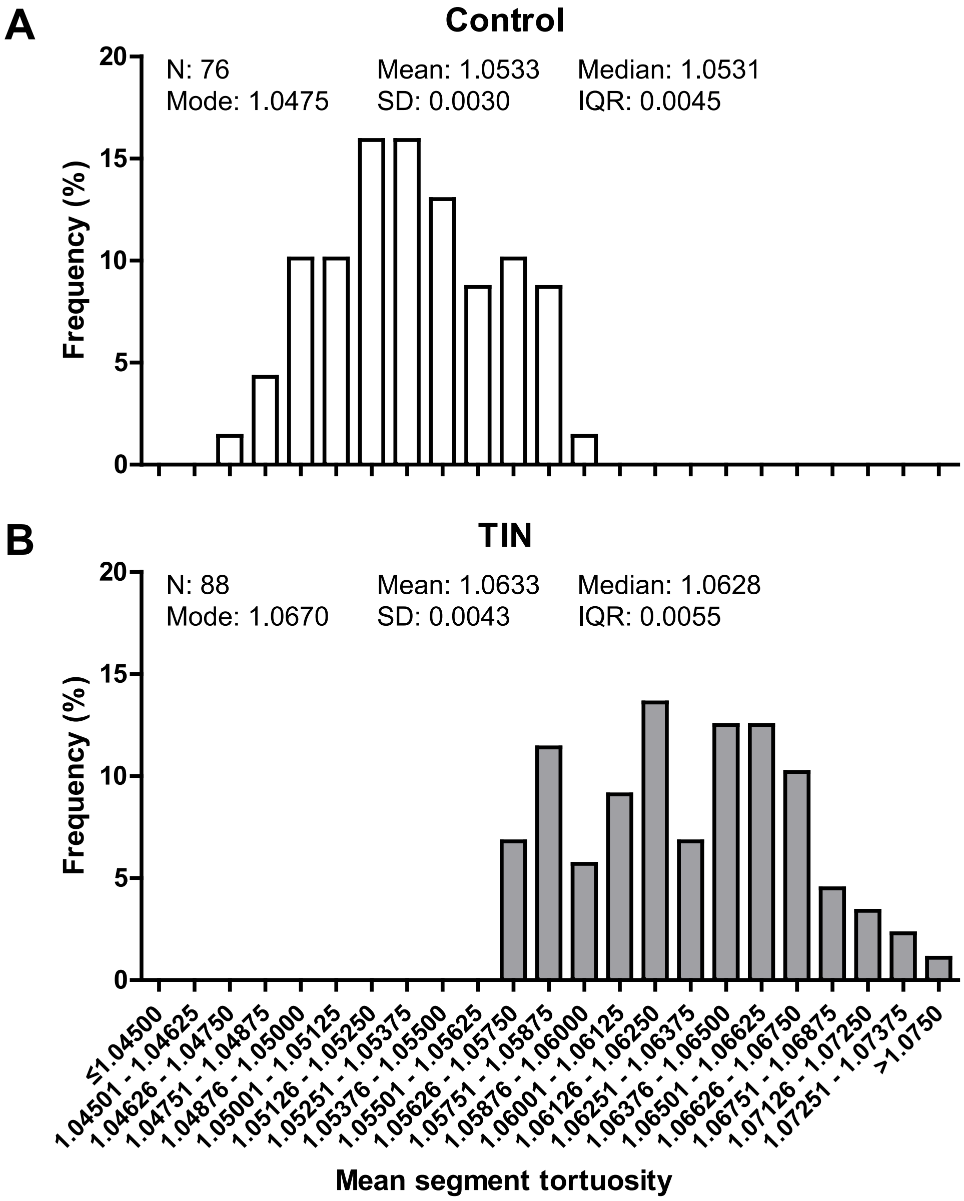

Supplementary Figure S6

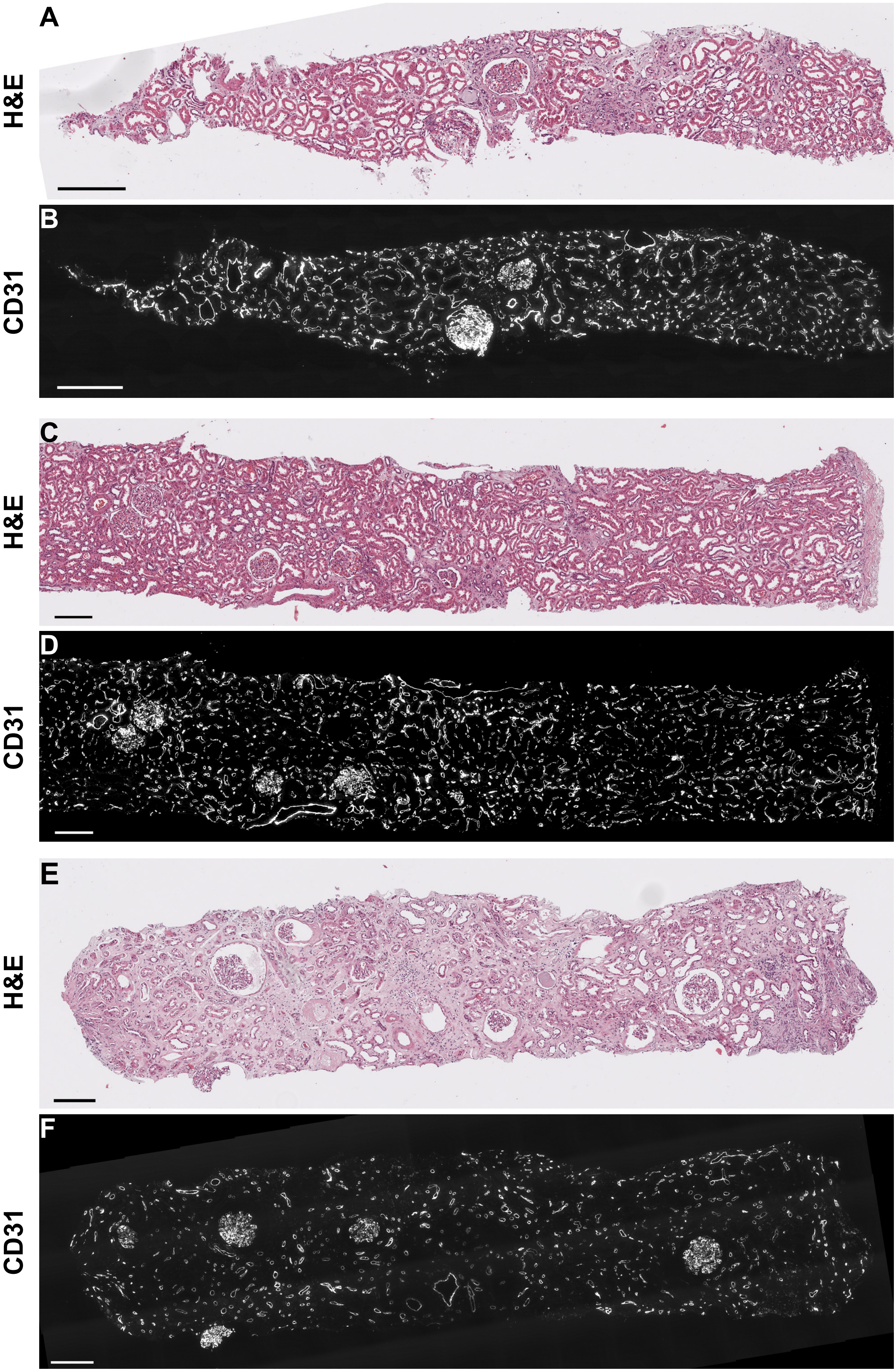

Supplementary Figure S7

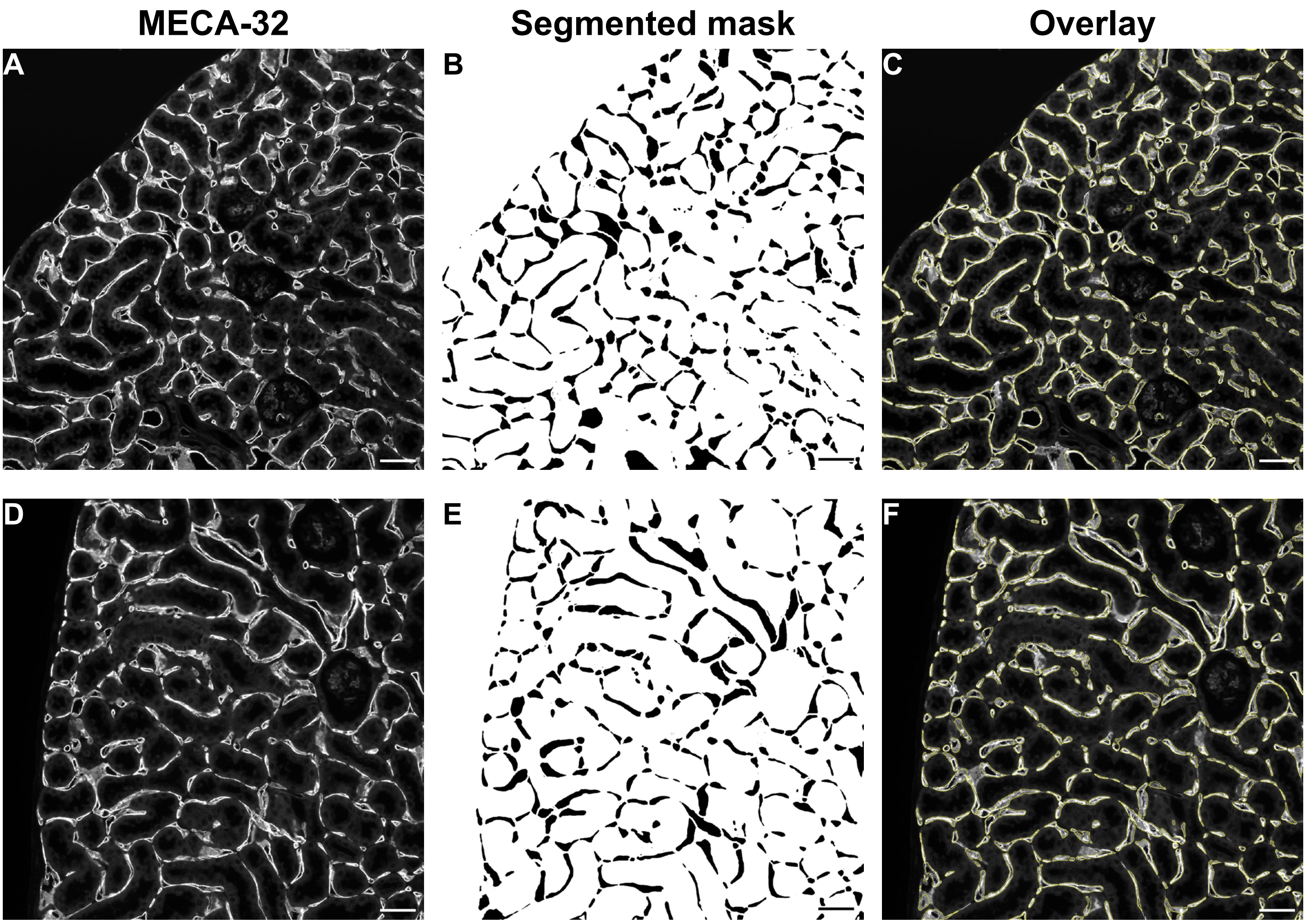

Supplementary Figure S8

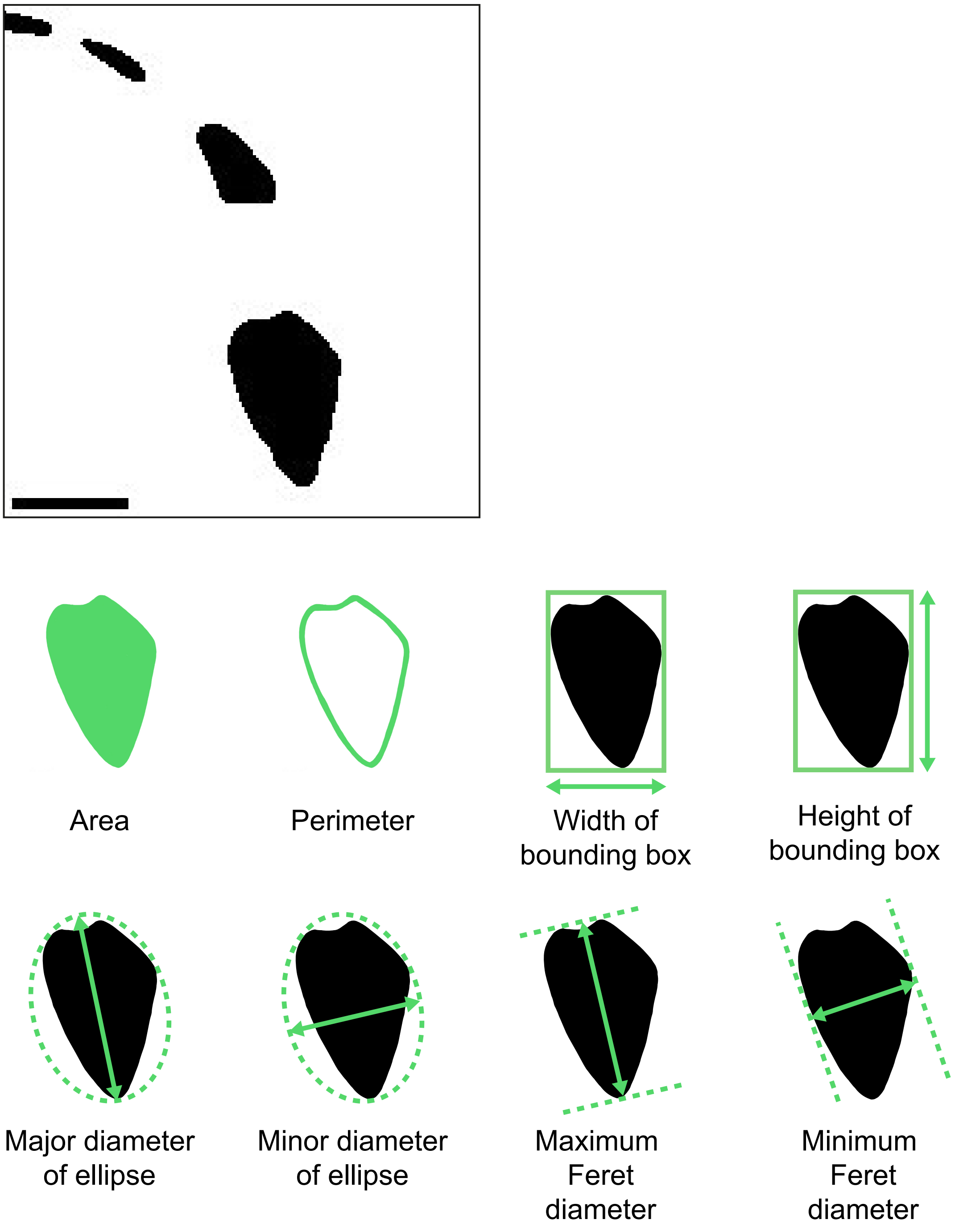

Supplementary Table S1: Morphometric evaluation of manually post-processed peritubular capillaries in mouse models with chronic tubulointerstitial injury.

Parameter	Control (n=14)	TIN (n=11)	UUO (n=10)	I/R (n=10)
Total area fraction (%)	16.2 ± 1.0	9.2 ± 0.7 *	12.4 ± 1.5 *	10.8 ± 1.6 *
Distance of neighbors (µm)	17.9 ± 0.6	26.4 ± 1.8 *	20.0 ± 1.4	21.9 ± 2.6 *
Luminal area (μm^2)	174.4 ± 12.3	126.7 ± 18.0	101.1 ± 8.9 *	86.2 ± 11.6 *
Perimeter (µm)	65.7 ± 2.5	43.6 ± 3.6 *	41.9 ± 1.9 *	38.5 ± 3.2 *
Width of bounding box (µm)	19.2 ± 0.8	13.2 ± 1.0 *	12.5 ± 0.6 *	11.2 ± 1.0 *
Height of bounding box (µm)	19.3 ± 0.8	12.9 ± 1.0 *	12.4 ± 0.6 *	11.8 ± 0.9 *
Maximum Feret diameter (µm)	26.2 ± 1.0	16.6 ± 1.3 *	16.3 ± 0.7 *	14.8 ± 1.2 *
Minimum Feret diameter (µm)	10.3 ± 0.3	9.0 ± 0.6	8.0 ± 0.3 *	7.9 ± 0.5 *
Major diameter of ellipse (µm)	23.5 ± 0.9	15.7 ± 1.2 *	15.3 ± 0.6 *	13.9 ± 1.1 *
Minor diameter of ellipse (µm)	8.3 ± 0.3	8.2 ± 0.5	7.1 ± 0.3 *	7.1 ± 0.4 *
Bounding box ratio	1.19 ± 0.04	1.12 ± 0.02 *	1.13 ± 0.04	1.04 ± 0.03 *
Feret ratio	2.65 ± 0.09	1.88 ± 0.04 *	2.05 ± 0.03	1.91 ± 0.06 *
Aspect ratio	3.06 ± 0.14	1.98 ± 0.05 *	2.23 ± 0.04	2.03 ± 0.08 *
Roundness	0.41 ± 0.01	0.57 ± 0.01 *	0.53 ± 0.01	0.56 ± 0.02 *
Circularity	0.52 ± 0.02	0.71 ± 0.02 *	0.67 ± 0.01	0.70 ± 0.02 *

Semiautomatically segmented and manually post-processed peritubular capillaries in the kidney cortex from control mice (n=14) and mice with adenine-induced tubulointerstitial nephritis (TIN, n=11), unilateral ureteral obstruction (UUO, n=10) and bilateral ischemia-reperfusion (I/R) injury (n=10) were evaluated morphometrically using Fiji/ImageJ. Mean values per mouse were used for statistical analysis.

Data are Mean±SD per group. * $p < 0.05$ Kruskal-Wallis H test followed by Dunn-Bonferroni *post hoc* test, in comparison with control.

Supplementary Table S2: Frequency of round peritubular capillaries in mouse models with chronic tubulointerstitial injury.

(A) Categorization of PTCs according to their roundness

	Absolute number per 10^5 µm²		Relative number	
	**Roundness <0.47**	**Roundness ≥0.47**	**Roundness <0.47**	**Roundness ≥0.47**
**Control**	61.1 ± 3.5	32.2 ± 3.6	65.6 ± 2.3	34.4 ± 2.3
**TIN**	22.8 ± 2.1*	51.1 ± 8.3	31.0 ± 2.4*	69.0 ± 2.4*
**UUO**	49.3 ± 5.6	73.6 ± 9.0*	40.1 ± 1.7	59.9 ± 1.7
**I/R**	42.6 ± 6.5*	84.1 ± 13.9*	33.8 ± 4.1*	66.2 ± 4.1*

(B) Categorization of PTCs according to their circularity

	Absolute number per 10^5 µm²		Relative number	
	**Circularity <0.60**	**Circularity ≥0.60**	**Circularity <0.60**	**Circularity ≥0.60**
**Control**	59.7 ± 3.9	33.6 ± 4.7	64.1 ± 3.7	35.9 ± 3.7
**TIN**	16.9 ± 3.1*	57.0 ± 9.6	23.1 ± 4.5*	76.9 ± 4.5*
**UUO**	40.1 ± 4.5	82.8 ± 10.6*	32.7 ± 2.1	67.3 ± 2.1
**I/R**	32.5 ± 6.1*	94.2 ± 14.1*	25.6 ± 3.9*	74.4 ± 3.9*

Semiautomatically segmented and manually post-processed peritubular capillaries in the kidney cortex from control mice and mice with adenine-induced tubulointerstitial nephritis (TIN), unilateral ureteral obstruction (UUO) and bilateral ischemia-reperfusion (I/R) injury were evaluated morphometrically. In ROC analysis the optimal cut-off values for roundness (0.47) and circularity (0.60) of PTCs to discriminate between chronic tubulointerstitial injury and control kidney were determined as the Youden index. The number of PTCs that were below or above the respective cut-off value was calculated for a standardized area (10^5 µm²) and relative to the total number of capillaries.

Data are Mean±SD per group. * $p < 0.05$ Kruskal-Wallis test followed by Dunn-Bonferroni *post hoc* test, in comparison with control.

Supplementary Table S3: Morphometric evaluation of unprocessed peritubular capillaries in mouse models with chronic tubulointerstitial injury.

**Parameter**	**Control (n=14)**	**TIN (n=11)**	**UUO (n=10)**	**I/R (n=10)**
Total area fraction (%)	16.9 ± 1.9	8.9 ± 1.1 *	11.6 ± 1.9 *	10.1 ± 1.7 *
Distance of neighbors (µm)	18.4 ± 0.5	26.6 ± 1.8 *	21.5 ± 1.8	22.7 ± 2.3 *
Luminal area (µm²)	259.0 ± 66.4	133.3 ± 23.0 *	119.0 ± 14.3 *	98.4 ± 20.1 *
Perimeter (µm)	96.6 ± 13.2	55.6 ± 4.4 *	52.1 ± 4.9 *	47.2 ± 5.6 *
Width of bounding box (µm)	23.5 ± 3.2	14.9 ± 1.2 *	14.1 ± 1.0 *	12.6 ± 1.5 *
Height of bounding box (µm)	23.5 ± 2.8	14.5 ± 1.0 *	14.0 ± 1.0 *	13.2 ± 1.3 *
Maximum Feret diameter (µm)	31.3 ± 3.8	19.2 ± 1.4 *	18.3 ± 1.3 *	16.7 ± 1.8 *
Minimum Feret diameter (µm)	13.8 ± 1.9	9.4 ± 0.7 *	9.0 ± 0.5 *	8.5 ± 0.9 *
Major diameter of ellipse (µm)	24.4 ± 2.7	16.7 ± 1.2 *	16.1 ± 0.9 *	14.8 ± 1.5 *
Minor diameter of ellipse (µm)	9.4 ± 1.2	7.6 ± 0.6 *	7.3 ± 0.4 *	7.0 ± 0.7 *
Bounding box ratio	1.18 ± 0.04	1.15 ± 0.02	1.13 ± 0.04 *	1.06 ± 0.04 *
Feret ratio	2.54 ± 0.08	2.07 ± 0.07 *	2.06 ± 0.06 *	1.98 ± 0.05 *
Aspect ratio	2.93 ± 0.12	2.29 ± 0.09 *	2.29 ± 0.08 *	2.19 ± 0.07 *
Roundness	0.43 ± 0.01	0.52 ± 0.02 *	0.52 ± 0.01 *	0.53 ± 0.01 *
Circularity	0.45 ± 0.01	0.59 ± 0.03 *	0.61 ± 0.02 *	0.61 ± 0.03 *

Semiautomatically segmented, not manually post-processed peritubular capillaries in the kidney cortex from control mice (n=14) and mice with adenine-induced tubulointerstitial nephritis (TIN, n=11), unilateral ureteral obstruction (UUO, n=10) and bilateral ischemia-reperfusion (I/R) injury (n=10) were evaluated morphometrically using Fiji/ImageJ. Mean values per mouse were used for statistical analysis.

Data are Mean±SD per group. * $p < 0.05$ Kruskal-Wallis H test followed by Dunn-Bonferroni *post hoc* test, in comparison with control.

Supplementary Table S4: Dimensions of idealized capillaries used in the computational model.

	**Shape of capillary cross section**	**Characteristic dimension (side, major axis, radius) (μm)**	**Capillary perimeter (μm)**	**Capillary cross-sectional area (μm^2)**	**Capillary density (%)**
**Physiological state**	Square	8.94	35.8	80.0	0.200
	Ellipse	7.14	34.6	80.0	0.200
	Circle	5.05	31.7	80.0	0.200
**Pathological state**	Square	7.90	31.6	62.5	0.100
	Ellipse	6.31	30.6	62.5	0.100
	Circle	4.46	28.0	62.5	0.100

Supplementary Table S5: Patient characteristics and histopathological findings in human kidney biopsies.

Patient number	Kidney disease	Sex	Age (years)	Serum creatinine (mg/dl)	eGFR (ml/min/ 1.73 m²)	GFR category	Protein-uria	IFTA (%)	IFTA (Grade)	Updated Oxford classification				
										M	E	S	T	C
1	Allograft 0h	M	86	0.90	80.0	2								
2	Allograft 3m	F	51	1.11	51.8	3	0.05 g/g	10-20	1					
3	Allograft 3m	M	75	1.49	46.0	3		10-20	1					
4	IgAN	M	50	0.80	102.3	1	6.0 g/d	<5	0	1	0	1	0	0
5	IgAN	F	38	0.60	111.9	1	0.9 g/d	<5	0	1	1	1	0	0
6	Allograft 3m	F	54	1.63	32.9	3		10-20	1					
7	IgAN	M	78	4.40	13.1	5	0.2 g/d	15	1	1	0	0	0	0
8	IgAN	M	59	6.00	9.7	5	5.5 g/d	50-60	3	1	0	1	2	1
9	IgAN	F	22	0.70	104.6	1	4.7 g/d	<5	0	1	1	1	0	1
10	IgAN	M	33	10.30	5.8	5		50-60	3	1	0	1	1	0
11	IgAN	M	62	5.50	10.6	5		60-70	3	1	0	1	2	0
12	Allograft 3m	F	30	1.99	29.4	4		10-20	1					
13	IgAN	M	52	0.90	88.6	2	2.0 g/d	<5	0	1	1	1	0	0
14	IgAN	M	37	0.90	95.0	1		<5	0	0	0	0	0	0
15	Allograft 3m	F	17	0.98	74.8	2	0.2 g/d	10-20	1					
16	Allograft 3m	M	17	1.40	66.8	2		<5	0					
17	Allograft 3m	F	45	1.50	37.6	3		10-20	1					
18	Allograft 3m	M	31	2.90	25.5	4		30	2					
19	IgAN	M	58	1.60	44.6	3		10-20	1	1	0	0	0	0
20	Allograft 3m	M	63	3.30	19.0	4	0.5 g/g	40-50	2					
21	IgAN	M	31	0.80	112.8	1	10.0 g/d	<5	0	1	0	1	0	0
22	IgAN	M	61	4.40	13.7	5	10.0 g/d	50-60	3	1	0	1	2	1
23	IgAN	M	73	1.50	45.9	3	0.8 g/g	10	1	1	0	0	0	1
24	IgAN	M	73	3.00	20.6	4	2.8 g/g	40-50	2	1	1	1	1	0
25	IgAN	F	26	1.70	36.3	3	10.0 g/d	20	1	1	1	1	1	1
26	IgAN	M	28	0.90	100.5	1	0.3 g/d	10	1	1	0	0	0	0

27	IgAN	M	75	6.10	9.0	5	9.6 g/d	30	2	1	0	0	1	1
28	IgAN	M	40	2.23	32.8	3		15	1	1	0	1	0	0
29	IgAN	M	68	1.40	50.4	3	0.1 g/g	15-20	1	1	0	0	0	0
30	IgAN	F	25	0.70	102.0	1		<5	0	1	0	0	0	1
31	IgAN	M	29	2.10	37.5	3	7.0 g/d	30-40	2	1	1	1	1	1
32	IgAN	M	41	1.80	41.8	3	1.0 g/d	20-25	1	1	0	1	0	1
33	IgAN	M	38	1.50	52.4	3	2.0 g/d	15	1	1	0	1	0	0
34	IgAN	M	55	1.30	57.3	3	0.1 g/d	10-15	1	1	1	0	0	0
35	IgAN	M	85	9.00	5.6	5		15-20	1	1	1	0	0	1
36	IgAN	M	47	1.70	43.4	3	5.0 g/d	20	1	1	1	1	0	1
37	IgAN	F	67	5.20	8.3	5	3.4 g/d	60	3					
38	IgAN	F	63	4.00	11.3	5		60	3					
39	IgAN	M	54	0.70	117.5	1	2.1 g/d	<5	0	1	1	1	0	0
40	IgAN	M	54	0.70	117.5	1	0.7 g/d	15	1	0	0	0	0	0

Abbreviations: IgAN=IgA nephropathy, allograft 0h=time-zero renal allograft, allograft 3m= renal allograft protocol biopsy at 3 months, eGFR=estimated glomerular filtration rate, IFTA=interstitial fibrosis and tubular atrophy, M=mesangial hypercellularity, E=endocapillary cellularity, S=segmental sclerosis, T=interstitial fibrosis/tubular atrophy, C=crescents.

GFR was calculated using the modified MDRD-4 equation and categorized according to the KDIGO classification (G1-G5). IFTA was scored semiquantitatively (0-3) according to the Banff classification. IgA nephropathy was evaluated using the MEST-C score of the updated Oxford classification. Proteinuria is given in g/d or g/g creatinine. The indicated parameters were obtained at the time of the biopsy.

Supplementary Table S6: Morphometric evaluation of peritubular capillaries in human kidney biopsies with chronic tubulointerstitial injury.

**Parameter**	**Control (n=27)**	**Chronic TI injury (n=37)**	
Total area fraction (%)	10.3 ± 3.2	7.4 ± 2.5	*
Luminal area (µm^2)	71.0 ± 63.3	45.0 ± 46.9	*
Perimeter (µm)	46.8 ± 15.6	31.0 ± 13.3	*
Width of bounding box (µm)	11.6 ± 4.1	8.2 ± 3.6	*
Height of bounding box (µm)	11.3 ± 3.5	8.0 ± 3.1	*
Maximum Feret diameter (µm)	15.1 ± 4.8	10.2 ± 4.1	*
Minimum Feret diameter (µm)	6.7 ± 2.2	5.6 ± 2.3	*
Major diameter of ellipse (µm)	12.8 ± 4.1	9.2 ± 3.8	*
Minor diameter of ellipse (µm)	5.0 ± 1.8	4.8 ± 2.1	
Bounding box ratio	1.18 ± 0.18	1.10 ± 0.12	
Feret ratio	2.37 ± 0.20	1.86 ± 0.13	*
Aspect ratio	2.75 ± 0.26	2.01 ± 0.17	*
Roundness	0.44 ± 0.04	0.57 ± 0.03	*
Circularity	0.41 ± 0.09	0.56 ± 0.10	*

Human kidney biopsies from renal allografts (n=10) and patients with IgA nephropathy (n=30) were examined. Within each biopsy, regions with preserved (control) tissue (n=27) and/or chronic tubulointerstitial (TI) injury (n=37) were manually selected. Semiautomatically segmented, unprocessed peritubular capillaries were analyzed morphometrically using Fiji/ImageJ. Mean values per biopsy and region were used for statistical analysis.

Data are Mean±SD per group. * $p < 0.05$ exact two-sided Mann-Whitney U test

Supplementary Table S7: Correlation of peritubular capillary morphology with kidney function and chronic tubulointerstitial injury in human patients.

Parameter	Serum creatinine	GFR	GFR category	IFTA score
Total area fraction (%)	-0.05	0.02	-0.06	-0.23
Luminal area (µm²)	-0.16	0.14	-0.20	-0.26
Perimeter (µm)	-0.48 *	0.48 *	-0.54 *	-0.45 *
Width of bounding box (µm)	-0.37 *	0.33 *	-0.41 *	-0.40 *
Height of bounding box (µm)	-0.28	0.28	-0.34 *	-0.31
Maximum Feret diameter (µm)	-0.35 *	0.32 *	-0.40 *	-0.37 *
Minimum Feret diameter (µm)	-0.29	0.30	-0.33 *	-0.31
Major diameter of ellipse (µm)	-0.24	0.19	-0.27	-0.29
Minor diameter of ellipse (µm)	-0.02	0.02	-0.03	-0.11
Bounding box ratio	-0.24	0.20	-0.23	-0.27
Feret ratio	-0.36 *	0.30	-0.38 *	-0.32
Aspect ratio	-0.37 *	0.33 *	-0.41 *	-0.32
Roundness	0.41 *	-0.36 *	0.43 *	0.35 *
Circularity	0.50 *	-0.53 *	0.58 *	0.39 *
Serum creatinine (mg/dl)	1.00	-0.98 *	0.96 *	0.84 *
GFR (ml/min/1.73 m²)	-0.98 *	1.00	-0.97 *	-0.83 *
GFR category	0.96 *	-0.97 *	1.00	0.85 *
IFTA score	0.84 *	-0.83 *	0.85 *	1.00

Morphology of human peritubular capillaries was correlated with established parameters of kidney function, i.e. serum creatinine, estimated glomerular filtration rate (GFR, according to the modified MDRD-4 equation) and GFR categories (G1-G5, according to the KDIGO classification), and chronic tubulointerstitial injury, i.e. interstitial fibrosis and tubular atrophy (IFTA) score (0-3, according to the Banff classification). Spearman correlation coefficients were calculated.

* $p < 0.05$ two-tailed t-test

Supplementary Table S8: Classification performance of peritubular capillary morphology for chronic kidney disease and tubulointerstitial injury in human kidney biopsies.

Parameter	AUC	95% CI
Total area fraction	0.453	0.244 - 0.662
Luminal area	0.567	0.360 - 0.773
Perimeter	0.790	0.629 - 0.951
Width of bounding box	0.673	0.487 - 0.860
Height of bounding box	0.677	0.482 - 0.871
Maximum Feret diameter	0.680	0.493 - 0.867
Minimum Feret diameter	0.667	0.478 - 0.855
Major diameter of ellipse	0.590	0.384 - 0.796
Minor diameter of ellipse	0.520	0.302 - 0.738
Bounding box ratio	0.547	0.344 - 0.750
Feret ratio	0.643	0.460 - 0.827
Aspect ratio	0.640	0.456 - 0.824
Roundness	0.660	0.476 - 0.844
Circularity	0.837	0.706 - 0.968

Peritubular capillaries in human kidney biopsies were evaluated morphometrically. Mean values of morphological parameters per total tissue samples were calculated. The ability of these parameters to classify chronic kidney disease (eGFR <60 ml/min/1.73 m^2 using the 4-variable MDRD equation) or interstitial fibrosis and tubulointerstitial injury (IFTA score >1 according to the Banff classification) was assessed by receiver operating characteristic (ROC) analysis. Data presented are the area under ROC curve (AUC) and 95% confidence intervals (CI).
